# AURKA suppresses NCOA4-mediated ferritinophagy to enhance sorafenib resistance in hepatocellular carcinoma

**DOI:** 10.1038/s41419-026-08774-2

**Published:** 2026-04-24

**Authors:** Wancui Zhu, Yilin Li, Zizhen Li, Jiajia Huang, Qiaohua Zhu, Huijuan Qiu, Enni Chen, Haohui Sun, Dingbo Shi, Miao Chen, Weining Xie, Wuguo Deng

**Affiliations:** 1 Sun Yat-sen University Cancer Center, State Key Laboratory of Oncology in South China, Guangdong Provincial Clinical Research Center for Cancer, Guangzhou, China; 2 Shunde Hospital of Southern Medical University, Foshan, Guangdong, China; 3 Guangdong Provincial Hospital of Integrated Traditional Chinese and Western Medicine; Affiliated Guangdong Hospital of Integrated Traditional Chinese and Western Medicine of Guangzhou University of Chinese Medicine; Nanhai Hospital of Traditional Chinese Medicine of Jinan University, Foshan, Guangdong, China

**Keywords:** Diseases, Cancer, Cancer therapy

## Abstract

Acquired resistance to sorafenib remains a major obstacle in the treatment of advanced hepatocellular carcinoma (HCC). While inducing ferroptosis represents a promising strategy to overcome this resistance, the specific molecular drivers underlying ferroptosis evasion in this context remain poorly defined. Here, we identified Aurora Kinase A (AURKA) as a central, actionable regulator of ferroptosis resistance in sorafenib-resistant HCC. AURKA was significantly upregulated in resistant cells and clinical specimens, which correlated with a suppressed ferroptotic state. Mechanistically, we discovered that AURKA directly interacted with and phosphorylated the ferritinophagy receptor NCOA4 at specific serine residues (S186/S234/S492), thereby competitively disrupting the NCOA4-FTH1 complex. This disruption inhibited ferritinophagic degradation of FTH1, stabilized the iron-storage protein, and limited the intracellular labile iron pool required for ferroptosis execution. Genetic or pharmacological inhibition of AURKA restored NCOA4-mediated ferritinophagy, synergized with ferroptosis inducers (sorafenib or IKE), and potently suppressed tumor growth both in vitro and in vivo. Clinically, high co-expression of AURKA and FTH1 predicted an unfavorable prognosis of HCC patients. Our study delineated the first direct link between AURKA kinase activity and the ferritinophagy machinery, establishing the AURKA-NCOA4-FTH1 axis as a master regulator of ferroptosis resistance in sorafenib-resistant HCC. These findings provide both a novel prognostic biomarker and a mechanistically grounded therapeutic strategy to overcome acquired resistance.

## Introduction

Liver cancer, predominantly hepatocellular carcinoma (HCC), stands as a leading cause of global cancer mortality with persistently poor survival rates [1]. Due to the insidious site and lack of early symptoms, most HCC patients are diagnosed at advanced stages, resulting in a low likelihood of curative surgical intervention [2]. Systemic therapy is the main therapeutic option for advanced HCC, and the immune checkpoint inhibitor-based immunotherapy have largely improved survival outcomes in some patients [3–5].

Sorafenib, a multi-targeted kinase inhibitor, remains a first-line treatment option for advanced HCC, especially for those unsuitable for or refractory to immunotherapy [2, 6, 7]. However, its efficacy is profoundly limited by acquired resistance [6]. Despite multi-dimensional investigations into the mechanisms of sorafenib resistance, current strategies are largely confined to preclinical genetic interventions [8–11]. The critical translational bottleneck persists due to the paucity of druggable molecular targets and specific pharmacological inhibitors [8]. Therefore, further exploration of novel resistance mechanisms and therapeutic strategies is critical for improving the prognosis of advanced HCC patients.

Ferroptosis, distinct from apoptosis, pyroptosis, and necroptosis, is an iron-dependent form of regulated cell death driven by lipid peroxidation and subsequent plasma membrane damage [12, 13]. Since the term was coined in 2012, ferroptosis has emerged as a research hotspot in the field of cancer development and progression [14–16]. Emerging evidence implicates ferroptosis resistance as a key contributor to sorafenib treatment failure, with restoration of ferroptosis sensitivity representing a promising therapeutic strategy [17–20]. Nevertheless, systematic elucidation of molecular mechanisms governing ferroptosis resistance remains elusive, hindering the development of targeted interventions. Iron homeostasis, which is regulated through uptake, export, storage, and release pathways, is essential for ferroptosis execution [16, 21–23]. Recent studies highlight ferritinophagy, a novel regulatory mechanism mediated by nuclear receptor coactivator 4 (NCOA4), as a critical modulator of iron metabolism and ferroptosis [24]. NCOA4 directly binds to ferritin heavy chain 1 (FTH1), the main iron storage protein, thereby releasing free iron ions. Paradoxically, NCOA4 depletion confers ferroptosis resistance by reducing intracellular iron availability [25–27]. Therefore, targeting ferritinophagy provides a novel strategy for inducing ferroptosis. However, its divergent roles across different tumor types underscore the need for further exploration of its molecular mechanisms and clinical translation [28–30].

Aurora Kinase A (AURKA), a serine/threonine kinase overexpressed in HCC and other cancers, is known to drive chemoresistance via classical pathways such as cell cycle regulation and apoptosis suppression [31–33]. Evidence supports that AURKA represents a promising target for cancer therapy [33, 34]. Some small molecules targeting AURKA have been developed. Alisertib (ALS, MLN8237) is an AURKA inhibitor that induces apoptosis by inhibiting the kinase activity of AURKA [35, 36]. ALS has been used in Phase II/Ⅲ clinical studies for various cancers. However, neither monotherapy nor combination therapy with traditional chemotherapy agents has yielded satisfactory therapeutic outcomes due to severe adverse effects, underscoring the need for new combination strategies [37–39]. Intriguingly, recent bioinformatics evidence reveals AURKA co-expression with ferroptosis-related genes in neuroblastoma, lung cancer, and head/neck squamous cell carcinoma, suggesting potential non-canonical role in ferroptosis regulation [40–42]. However, whether AURKA mediates sorafenib resistance by modulating ferroptosis in HCC remains unknown. Addressing this knowledge gap could provide new theoretical foundations for combination therapies.

In this study, we uncovered for the first time that AURKA overexpression drove ferroptosis resistance in sorafenib-resistant HCC. Genetic depletion of AURKA potently sensitized resistant cells to sorafenib and ferroptosis inducers (e.g., RSL3), which was accompanied by elevated reactive oxygen species (ROS) and lipid peroxidation. Mechanistically, AURKA competitively bound to NCOA4 in a kinase-dependent manner, thereby disrupting the NCOA4-FTH1 complex. This interference inhibited NCOA4-mediated autophagic degradation of FTH1—ferritinophagy, leading to stabilization of FTH1, sequestering of intracellular iron, and suppressing of lipid peroxidation, ultimately conferring ferroptosis resistance. Pharmacological inhibition of AURKA restored NCOA4-FTH1 binding and synergized with ferroptosis inducers to overcome therapeutic resistance in vitro and in vivo. Clinically, elevated co-expression along the AURKA-FTH1 axis correlated with poor prognosis in HCC patients. These findings not only elucidate a novel resistance mechanism but also provide a translational framework for optimizing AURKA inhibitor-based combination therapies.

## Materials and methods

### Cell lines

The human HCC cell line HepG2 was provided by Professor Minshan Chen at Sun Yat-sen University Cancer Center (Guangzhou). The Huh7 cell line was purchased from China National Center for Cell Science (Shanghai). HEK293T cells were purchased from the American Type Culture Collection (ATCC). The sorafenib-resistant cell lines HepG2 SR and Huh7 SR were established via concentration gradient induction. All cell lines were authenticated by short tandem repeat (STR) profiling within the last three years and were confirmed to be mycoplasma-free using PCR-based detection methods. Cells were maintained in DMEM medium (Corning), with the addition of 10% fetal bovine serum (FBS; ExCell Bio) and 100 U/ml penicillin-streptomycin at 37 °C in a humidified incubator with 5% CO2.

### Reagents and antibodies

Alisertib (S1133), CD532 (E1651), RSL3 (S8155), IKE (S8877), Ferrostatin-1 (S7243), Defer-oxamine mesylate (S5742), and Sorafenib (S7397) were bought from Selleck (Shanghai, China). The primary antibody against GAPDH (60004-1-Ig), Flag tag (20543-1-AP), MYC tag (16286-1-AP), HA tag (66006-2-Ig), AURKA (66757-I-Ig) for IF, NCOA4 (10968-I-Ap) for IF were bought from Proteintech; AURKA (91590S), DMT1 (15083S), NCOA4 (66849S), PTGS2 (12282 T), FTH1 (4393 T) for IHC, Phospho-AURKA (Thr288) (3079 T) were purchased from Cell Signaling Technology; TFRC1 (R25971) was bought from Zen BioScience; FTH1 (sc-376594) for western blot was bought from Santacruze Biotechnology; Ferritin (ab75973) for IF and Ki67 (ab15580) were bought from Abcam; Dylight488 (E032210) and Dylight594 (E032420) for IF were bought from Earthox.

### RNA extraction and RT-qPCR

Total RNA was extracted with the Cell RNA Rapid Extraction Kit from GOONIE (400-100-100 T) according to the manufacturer’s instructions. Complementary DNA (cDNA) was synthesized using Fast Reverse Transcription kit from Vazyme (RT101-01). The qRT-PCR was carried out on a CFX96 Touch sequence detection system (Bio-Rad) or a LightCycler 480 System (Roche), with HisyGo RT Red SuperMix from Vazyme (RT101-01). The specific primers were synthesized by Ruibo Biotechnology Company (Guangzhou, China) and the detailed sequences were as follows: GAPDH, forward 5′-CTGACTTCAACAGCGACACC-3′, reverse 5′-TGCTGTAGCCAAATTCGTTGT-3′; AURKA, forward 5′-GAGGTC

CAAAACGTGTTCTCG-3′, reverse 5′-ACAGGATGAGGTACACTGGTTG-3′; FTH1, forward 5′-CTTTGACCGCGATGATGTGG-3′, reverse 5′-CCTGAAGGAAGAT

TCGGCCA-3′; PTGS2, forward 5′-GTTCCACCCGCAGTACAGAA-3′, reverse 5′-

AGGGCTTCAGCATAAAGCGT-3′. The expression levels of target genes were nor-malized by GAPDH.

### Western blot analysis

Protein was extracted from the cells using RIPA lysis buffer (Beyotime, P0013B) supplemented with protease and phosphatase inhibitors (Beyotime, P1045), separated by SDS-polyacrylamide gels, and then transferred to PVDF membranes from Merck Millipore (IPVH00010). The membranes were blocked with 5% bovine serum albumin and incubated with primary antibodies overnight. After that, a peroxidase-conjugated secondary antibody was applied to the membranes. The antigen-antibody interaction was visualized using an enhanced chemiluminescence assay (ECL, Zen BioScience, 17046).

### Co-Immunoprecipitation

After the indicated treatments, cells plated in a 10-cm dish were lysed in 500 μL lysis buffer (Beyotime, P0013) supplemented with protease and phosphatase inhibitors (Beyotime, P1045) for 30 min on ice. After centrifugation at 13,000 rpm for 15 min, the supernatants were collected to measure the protein concentration by using the BCA Protein Assay Kit (Beyotime, P0010) according to the guidelines. 100 μg protein was saved for input. An equivalent quantity (1000 μg) of protein was immunoprecipitated with the specific antibody or IgG control overnight at 4 °C. The next morning, add 30 μL slurry volume of Protein G magnetic beads (MCE, HY-K0202) to each sample to incubate for 4 h. Afterward, the precipitants were washed 3 times (5 min per time) with washing buffer A (50 mM Tris-HCl, pH 7.5,0.5% Triton X-100, 150 mM NaCl, 2 mM CaCl2, 5% glycerol, 2 mM PMSF), 1 time (5 min) with washing buffer B (50 mM Tris-HCl, pH 7.5, 0.5% Triton X-100,500 mM NaCl, 2 mM CaCl_2_, 5% glycerol, 2 mM PMSF), and 2 times (5 min per time) with washing buffer C (50 mM Tris-HCl, pH 7.5, 150 mM NaCl, 2 mM CaCl_2_). After the final wash, the supernatant was removed and the immune complexes were eluted with 1 x loading buffer for 5 min at 100°C. The input and IP fractions were analyzed via SDS-PAGE and western blot.

### Assessment of cytosolic ROS

HCC cells were seeded in 12-well plates and treated as indicated. Then, cells were incubated with 0.5 μM ROS detection probe DCFH-DA (Solarbio, 4091-99-0) for 30 min at 37°C in the dark. Then unincorporated dye was removed by washings with PBS. Samples were then digestion by trypsin and centrifuged at 1000 rpm for 3 min and the pellets were resuspended in PBS. The signals (FITC channel) were measured on a flow cytometer (cytoFLEX LX, Beckman Coulter, America) by analyzing 10,000 cells with the CytExpert software.

### Lipid peroxidation assay

The lipid peroxidation assay was performed with BODIPY 581/591 C11 (27086) from Cayman Chemical according to the manufacturer’s instructions. HCC cells were seeded in 12-well plates and cells were treated as indicated. Then, cells were incubated with 1 μM BODIPY 581/591 C11 at 37°C for 30 min in the dark. Then the cells were collected at 1000 rpm for 3 min and resuspended in 500 μL PBS for flow cytometry analysis. Oxidized C11 (FITC channel) was measured on a flow cytometer (cytoFLEX LX, Beckman Coulter) by analyzing 10,000 cells with the CytExpert software. For observation using a fluorescence microscope, the samples were incubated with BODIPY 581/591 C11 probe for 30 min, the staining solution was then removed, 500 μL PBS was added, and the samples were subsequently observed and imaged in FITC (oxidized state) and Cy3 (reduced state) channels.

### Cellular ferrous iron detection

The levels of cellular ferrous iron were assessed by FerroOrange probe (F374) from Dojindo following the instructions provided. Briefly, to detect the cellular ferrous iron, cells were treated as indicated. Then cells (1×10^4^ /well) were seeded in black transparent bottom 96-well and grown overnight. The supernatant was discarded and cells were washed three times with serum-free media, followed by incubated with 0.5 μM FerroOrange probe at 37 °C for 20 min. The fluorescence intensity of ferrous ions was measured using a TECAN (Spark 10 M, America) multifunctional microplate reader (Ex:543 nm/Em:580 nm).

### Cell viability assay

Cell viability measurement was performed using the Cell Counting Kit-8 (CCK-8) assay. Briefly, cells (3000/well) were seeded in a 96-well plate. About 12 h after cell seeding, cells were pre-treated with indicated compounds at the indicated concentrations for the indicated time. There were three biological replicates per condition. After treated, RPMI 1640 media (100 μL) containing 10 μL CCK-8 reagent (GLPBIO, GK10001) was added to each well and the 96-well plate was incubated at 37°C for 1 h in the dark. The absorbance of each well was detected at 450 nm by using a Multiskan Spectrum Microplate Spectrophotometer (MD VersaMax, America). Relative cell viability to the control group was calculated.

### Short interfering RNAs transfection

The short-interfering RNA (siRNA) molecules were synthesized by GenePharma Biotechnology (Shanghai, China). The transfection of siRNA was carried out using Lipofectamine™ RNAiMAX Transfection Reagent (ThermoFisher, 13778) following the guidelines and instructions of manufacturers. The targeting sequence to NCOA4 was 5′-UGAACAGGUGGACCUUAUUUA-3.

### Protein purification and in vitro kinase assay

The recombinant human His-tagged AURKA protein was purchased from abinScience (HT394012). The HA-tagged wild-type (WT) NCOA4 protein and its phosphorylation-site mutant (S186A/S234A/S492A) were purified from HEK 293 T cells using HA-tag fusion protein purification kit (DIA-AN, KAP0063). Western blot was performed to verify the purity and molecular weight of the recombinant protein. In vitro kinase assay, the reaction was performed in a final volume of 50 μL. Briefly, purified AURKA kinase (500 ng) was incubated with purified wild-type or mutant NCOA4 substrate (5 μg) in 1× kinase reaction buffer supplemented with 10 μM ATP. The reaction mixture was gently mixed and then incubated at 30 °C for 30 min. The reaction was terminated by adding an equal volume (50 μL) of 2× SDS-PAGE loading buffer, followed by boiling at 100°C for 5 minutes. Phosphorylation of NCOA4 was subsequently analyzed by Western blot.

### Immunofluorescence

Cells were treated according to the experimental design, digested, and seeded into confocal dishes at a density of ~30%. After cells adhered to the dishes, wash cells with PBS and add 200 μL of 4% paraformaldehyde to fix at room temperature (RT) for 15 min, then wash with PBS twice. Add 200 μL of 0.3% Triton X-100 (diluted in PBS) to permeabilize at RT for 20 min and then wash with PBS three times. Add 100 μL of 5% goat serum (diluted in PBS) to cover the cells, block at RT for 60 min. Remove blocking solution and add 100 μL of primary antibody diluted in 5% goat serum to incubate at 4°C overnight (AURKA: 1:200, NCOA4: 1:200, LAMP1: 1:100, Ferritin: 1:100). The next day, wash with PBST three times and add secondary antibodies diluted in 5% goat serum (DyLight 594 Goat Anti-Rabbit: 1:400; DyLight 488 Goat Anti-Mouse: 1:400), incubate at RT protected from light for 1 h, then wash with PBST three times. Add 100 μL DAPI and incubate at RT protected from light for 5 min, then wash with PBST five times. Observe fluorescence using FITC488, 594, and DAPI channels under a 63× objective lens.

### Immunohistochemistry

The tissue microarray, consisting of 134 HCC specimens, was obtained from the Sun Yat-sen University Cancer Center, with approval from the center’s Ethics Committee (Approval No. SL-B2022-724-01). Paraffin-embedded tissue samples were cut into sections of 3 μm thickness. The specimens were incubated with primary antibodies against AURKA (1:200), FTH1 (1:200), PTGS2 (1:500) and Ki-67 (1:200) overnight at 4 °C. Immunodetection was performed the next day using DAB (Vector, SK-4100) following the manufacturer’s instructions. Images of the stained samples were captured using Jiangfeng Automatic Slide Scanner. IHC staining of AURKA, FTH1, Ki-67, and PTGS2 were scored independently using the IRS (Immunoreactive Score) system to ensure consistency and accuracy. The intensity of staining was categorized into four scores: 0 (no brown particle staining), 1 (light brown particles), 2 (moderate brown particles), 3 (dark brown particles). The percentage of positive tumor cells was classified into four scores: 1 ( < 10% positive cells), 2 (10–50% positive cells), 3 (51–80% positive cells), and 4 ( > 80% positive cells). The IRS was determined by multiplying the two scores together, resulting in a range of scores from 0 to 12.

### Animal experiments

Animal experiments in this study were approved by the Experimental Animal Ethics Committee, Sun Yat-sen University Cancer Center (L102042023050L). All experiments were performed in accordance with the approved guidelines and regulations. 4-week-old male BALB/c-Nude mice were purchased from Guangdong Gempharmatech (Foshan, China). The mice were subsequently housed at the Animal Experiment Center of Sun Yat-Sen University. BALB/c-Nude mice were subcutaneously injected with WT or AURKA knocked-down Huh7 SR cells at a density of 2 × 10^7^ cells. When tumors grew to about 100 mm^3^, a simple randomization procedure (drawing lots) was used to allocate animals to experimental groups. Mice were assigned to one of three groups treated with 30 mg/kg sorafenib via gavage every day, 30 mg/kg IKE administered by intraperitoneal injection every day, or saline as a control, and the mice were sacrificed on day 22. The weights of the excised tumors were documented and recorded. Tumor dimensions were measured every 2 days by a digital caliper. For the AURKA inhibitor combination sorafenib or IKE, 20 mg/kg ALS was administered by intraperitoneal injection every day. To ensure objectivity, the investigator performing tumor measurements and data analysis was blinded to the group allocation.

### Public databases and cellular sequencing data analysis

Transcriptomic data and clinical information for the TCGA-LIHC (The Cancer Genome Atlas Liver Hepatocellular Carcinoma) cohort were obtained from the GDC Data Portal (https://portal.gdc.cancer.gov/). Survival analysis was performed using the survival package in R to construct survival models, with Kaplan-Meier curves generated via the survminer package. Intergroup survival differences were statistically evaluated using the Log-Rank test. The GEO datasets GSE121153 and GSE25097 were downloaded from the NCBI GEO database (Gene Expression Omnibus, https://www.ncbi.nlm.nih.gov/geo/). Differential gene expression analysis was conducted using the limma package. The mRNA sequencing data from HepG2/Huh7 sorafenib-resistant and sensitive cells were normalized with FPKM (Fragments Per Kilobase Million) and analyzed for differential expression using the edgeR package. Threshold criteria were defined as follows: upregulated genes (log2FC ≥ 0.5, *P* < 0.05) and downregulated genes (log2FC < -0.5, *P* < 0.05).

### Quantification and statistical analysis

For quantitative analysis presented as histograms, values were obtained from at least three independent experiments and were presented as the mean ± SD. For comparisons between two groups, two-tailed unpaired Student’s t test or two tailed Mann–Whitney test was used. For comparisons involving more than two groups, one-way ANOVA followed by Tukey multiple comparisons test was applied. For time-course or grouped data, two-way ANOVA was used. Correlation analysis was performed using Pearson correlation coefficient. Survival curves were generated using the Kaplan-Meier method and compared using the log-rank test. A *P* value < 0.05 was considered statistically significant. All statistical analyses were performed with GraphPad Prism version 8.0 software.

## Results

### Sorafenib-resistant HCC cells exhibit lower ferroptosis activity

**Fig. 1:**
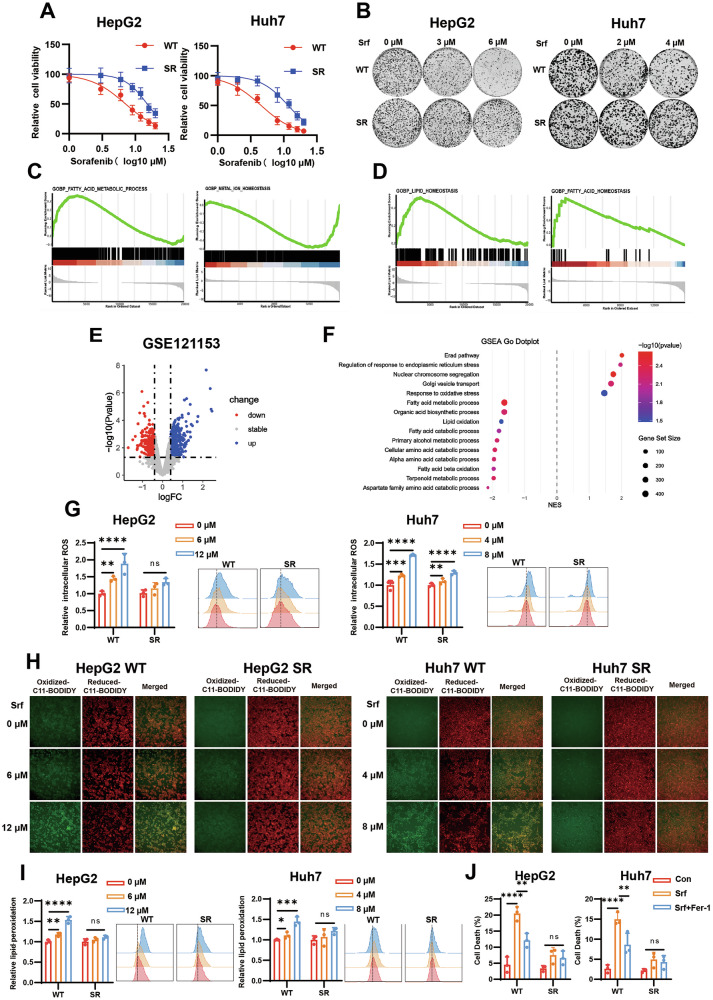
Sorafenib-resistant HCC cells exhibit lower ferroptosis activity. **A** CCK-8 assay assessing proliferative activity of wild-type (WT) and sorafenib-resistant (SR) HCC cells after 48 h treatment with sorafenib (Srf). **B** Colony formation assay demonstrating the colony-forming ability of wild-type (WT) cells and sorafenib-resistant (SR) cells treated with sorafenib for 48 h. **C**, **D** Gene Set Enrichment Analysis (GSEA) of ferroptosis-related pathways in HepG2_SR vs HepG2_WT (**C**) and Huh7_SR vs Huh7_WT (**D**). **E** Volcano plots of differentially expressed genes in sorafenib-resistant (*n* = 5) vs sensitive tissues (n = 3) from RNA sequencing in GSE121153 dataset. (DEGs, |log2FC | > 0.5, *P* < 0.05). **F** GSEA enrichment results of DEGs in GSE121153. **G** Detection of the intracellular ROS levels in SR and WT cells treated with sorafenib for 48 h. **H**, **I** Fluorescence microscopy images (**H**) and Flow cytometry (**I**) showing the fluorescence intensity of lipid peroxidation labeled with BODIPY-C11 probe (1 μM) in SR and WT cells after 48 h exposure. **J** Cell death in WT and SR cells treated with sorafenib for 48 h, following pretreatment with ferrostatin-1 (Fer-1, 5 µM). Data in (**A**, **G**, and **I, J**) are representative of three independent experiments and presented as mean ± S.D. Statistical analysis was performed by one-way ANOVA with Tukey multiple comparisons test (**G**, **I, J**). (ns *P* ≥ 0.05, * *P* < 0.05, ** *P* < 0.01, *** *P* < 0.001, **** *P* < 0.0001).

To elucidate the possible mechanisms underlying sorafenib resistance, we established sorafenib-resistant cell lines (HepG2 SR and Huh7 SR) via concentration gradient induction and demonstrated that these drug-resistant HCC cell lines had significantly increased half-maximal inhibitory concentration (IC50) values compared to the parental cells (Fig. 1A). Specifically, the IC50 of HepG2 SR cell line increased from 7.11 ± 0.90 μM in parental cells to 15.25 ± 1.56 μM, while the IC50 of Huh7 SR cell line increased from 4.56 ± 0.44 μM in parental cells to 11.66 ± 1.21 μM (Fig. 1A). Colony formation assays further validated the stability of the resistant phenotype. Under sorafenib treatment, resistant cells maintained proliferative capacity, whereas the growth of parental cells was markedly suppressed, confirming phenotypic stability (Fig. 1B and Fig. S1A). Together, these findings confirmed the successful establishment of the sorafenib-resistant HCC cell lines.

To further explore the molecular mechanisms underlying acquired sorafenib resistance in HCC cells, we performed mRNA sequencing on wild-type sensitive cells (HepG2 WT and Huh7 WT) and their resistant counterparts (HepG2 SR and Huh7 SR). Comparative analysis revealed 688 significantly and differentially expressed genes (DEGs) in HepG2 SR versus HepG2 WT, and 590 DEGs in Huh7 SR versus Huh7 WT (Fig. S1B). Subsequent single-sample gene set enrichment analysis (ssGSEA) demonstrated significant enrichment of ferroptosis-related signaling pathways, including fatty acid homeostasis, lipid homeostasis, and metal ion homeostasis (Fig. 1C, D). Additionally, analysis of a sorafenib resistance dataset from HCC (GSE121153) revealed conserved activation of ferroptosis-associated pathways, particularly those involved in fatty acid metabolism, ROS stress, and lipid oxidation (Fig. 1E, F). These findings provided critical insights into the potential link between sorafenib resistance and ferroptosis evasion in HCC cells.

To clarify the association between sorafenib resistance and ferroptosis inhibition, we systematically analyzed ferroptotic responses in resistant (SR) and wild-type (WT) HCC cells under escalating sorafenib concentrations. Using fluorescent probes DCFH-DA (for ROS) and BODIPY-C11 (for lipid peroxidation), we observed significant accumulation of ROS (Fig. 1G) and lipid peroxidation (Fig. 1H, I) in parental cells, whereas SR cells exhibited no significant changes. Consistently, cell death analysis by propidium iodide (PI) staining confirmed that sorafenib induced significant death in WT cells, which was specifically rescued by the ferroptosis inhibitor ferrostatin-1 (Fer-1). In contrast, SR cells were resistant to sorafenib-induced cell death (Fig. 1J). This resistance phenotype, aligned with the transcriptomic findings, confirmed that ferroptosis evasion is a critical adaptive mechanism underlying acquired sorafenib resistance in HCC.

### AURKA overexpression drives ferroptosis resistance

**Fig. 2:**
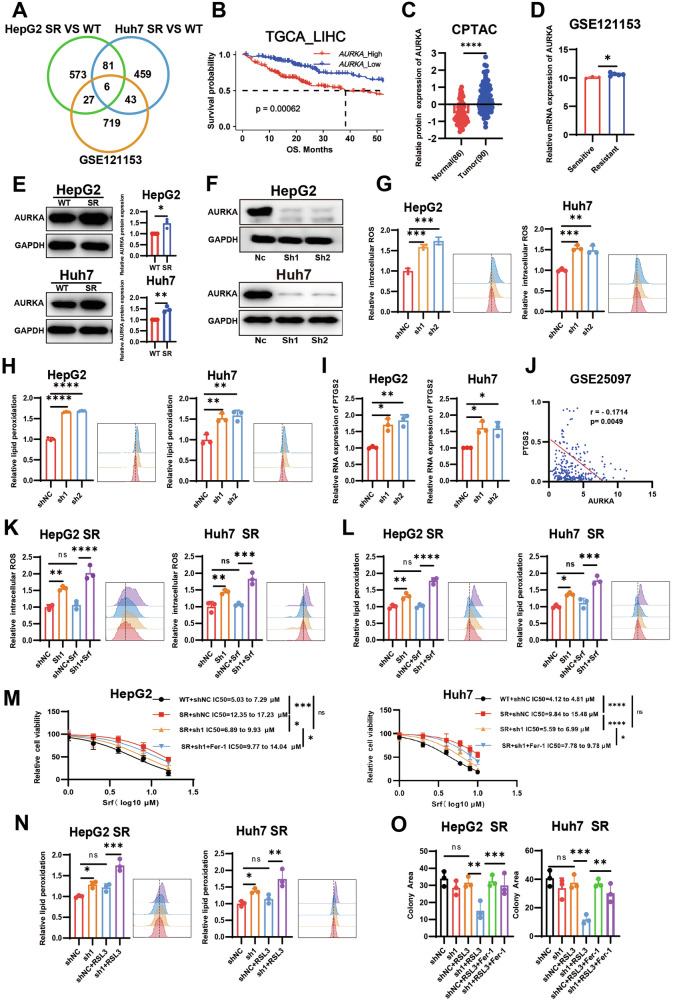
AURKA overexpression drives ferroptosis resistance. **A** Venn diagram showing the overlapped DEGs from transcriptomic analysis of SR vs WT HepG2/Huh7 cells and the GSE121153 dataset. **B** Kaplan-Meier survival analysis demonstrates the relationship between AURKA mRNA expression levels and overall survival in TCGA-LIHC patients. **C** CPTAC proteomics data showing AURKA protein in HCC (n = 90) vs adjacent normal tissues (n = 86). **D** AURKA mRNA expression in sorafenib-resistant (n = 5) vs sensitive (n = 3) tissues in GSE121153 dataset. **E** Western blot and qualitative analysis of AURKA protein expression levels in SR and WT cells. **F** AURKA knockdown validation in HCC cells transduced with Lv-shAURKA vs Lv-NC. **G, H** Detection of the intracellular ROS levels (DCFH-DA probe, 0.5 µM) (**G**) and lipid peroxidation (BODIPY-C11 probe, 1 µM) (**H**) levels post-AURKA knockdown. **I** PTGS2 mRNA expression by qRT-PCR in HepG2 and Huh7 cells after AURKA knockdown. **J** Correlation between AURKA and PTGS2 mRNA expression levels in the GSE25097 dataset. **K**, **L** Detection of the intracellular ROS levels (**K**) and lipid peroxidation levels (**L**) in AURKA-knockdown SR HCC cells treated with sorafenib (5 µM) for 48 h. **M** CCK-8 assay showing restored sorafenib sensitivity in AURKA-knockdown SR cells reversed by ferrostatin-1 (Fer-1, 5 µM). **N** Detection of the lipid peroxidation levels with BODIPY-C11 probe in AURKA-knockdown SR HCC cells treated with RSL3 (HepG2:0.5 µM; Huh7:100 nM) for 48 h. **O** Quantitative analysis of the colony formation assay. Data in (**G**, **H**, **I**, **K**, **L**, **and N**, **O**) are representative of three independent experiments and presented as mean ± S.D. Statistical analysis was performed by Kaplan-Meier method with the log-rank test (**B**), two tailed Mann–Whitney test (**C**), two-tailed unpaired t test (**D**, **E**), two-way ANOVA (**M**), one-way ANOVA with Tukey multiple comparisons test (**G**, **H**, **I**, **K**, **L**, **N**, **O**) and Pearson correlation analysis (**J**). (ns *P* ≥ 0.05, * *P* < 0.05, ** *P* < 0.01, *** *P* < 0.001, **** *P* < 0.0001).

To elucidate the molecular regulatory network underlying ferroptosis resistance in sorafenib-resistant HCC cells, we conducted an integrated analysis of DEGs from sorafenib-resistant cells (HepG2-SR vs. HepG2-WT, Huh7-SR vs. Huh7-WT) and the GSE121153 dataset. Six overlapping DEGs (*AURKA, DUSP5, LIPH, MYOF, RND3*, and *GRB10*) were identified (Fig. 2A). Subsequent Kaplan-Meier survival analysis using the TCGA-LIHC cohort revealed that elevated AURKA expression was uniquely associated with significantly shorter overall survival (OS) in HCC patients (Fig. 2B and Fig. S2A–E). Proteomic analysis of the CPTAC database demonstrated significantly higher AURKA protein expression in HCC tissues versus adjacent normal tissues (Fig. 2C). Transcriptomic validation using the GSE121153 dataset confirmed elevated AURKA mRNA levels in sorafenib-resistant tissues (Fig. 2D). Additionally, western blot analysis demonstrated substantial upregulation of AURKA protein in sorafenib-resistant cells compared to their parental counterparts (Fig. 2E). These findings collectively suggested that AURKA may serve as a potential regulatory factor driving sorafenib resistance in HCC.

As a core regulator of the cell cycle, AURKA governs genomic stability by modulating centrosome maturation, spindle assembly, and chromosome segregation [43, 44]. Notably, its aberrant overexpression in HCC drives tumor progression and therapy resistance by activating multiple oncogenic signaling pathways, including PI3K/AKT and NF-κB, thereby promoting tumor cell proliferation, invasion, metastasis, and epithelial-mesenchymal transition (EMT) [32, 45, 46]. Emerging evidence from neuroblastoma and head neck squamous cell carcinoma (HNSCC) models suggests AURKA co-expresses with ferroptosis-related genes [40, 41]. To identify whether a similar mechanism operates in HCC, we established stable AURKA-knockdown models in both WT and SR cells, with knockdown efficiency confirmed by western blot (Fig. 2F and Fig. S2F). Compared with controls, AURKA-knockdown significantly increased ROS accumulation and lipid peroxidation in WT HepG2 and Huh7 cells (Fig. 2G, H). Correspondingly, mRNA expression of the ferroptosis biomarker PTGS2 was upregulated (Fig. 2I). Analysis of the GSE25097 dataset corroborated this finding, revealing a significant negative correlation between AURKA and PTGS2 mRNA levels in HCC tissues (Fig. 2J). Furthermore, in these wild-type cells, AURKA knockdown significantly sensitized them to both sorafenib and the ferroptosis inducer RSL3 (Fig. S2G–L). Together, these multi-dimensional results indicated that AURKA acts as a negative regulator of ferroptosis in HCC.

We then investigated whether AURKA contributes to ferroptosis resistance in SR cells. Although SR cells showed significant resistance to sorafenib-induced ferroptosis, AURKA knockdown markedly enhanced sorafenib-induced ROS accumulation (Fig. 2K and Fig. S2F) and lipid peroxidation levels (Fig. 2L). Consistently, CCK-8 assays demonstrated that AURKA knockdown significantly resensitized SR cells to sorafenib, an effect that was reversed by the ferroptosis inhibitor ferrostatin-1 (Fer-1) (Fig. 2M). Moreover, HepG2 SR and Huh7 SR cells were also resistant to ferroptosis inducer RSL3, and AURKA knockdown restored their sensitivity to RSL3 (Fig. 2N, O and Fig. S2M). These data suggested that AURKA mediates broad ferroptosis resistance in SR cells.

Collectively, these results demonstrated that the elevated expression of AURKA in sorafenib-resistant HCC cells drive ferroptosis resistance and acquired drug tolerance. The consistent sensitizing effect of AURKA knockdown in both WT and SR cells underscored its central role in regulating ferroptotic sensitivity in HCC.

### AURKA knockdown sensitizes sorafenib-resistant tumors to ferroptosis inducers in vivo

**Fig. 3:**
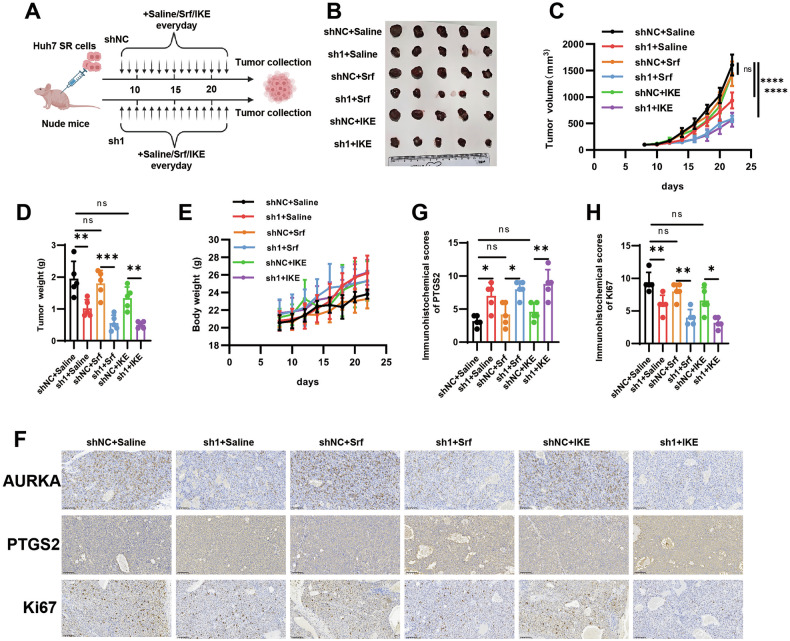
AURKA knockdown sensitizes sorafenib-resistant tumors to ferroptosis inducers in vivo. **A** Schematic diagram of tumor inoculation and treatment in mice. Arrows indicate the exact days for IKE, sorafenib, or saline administration. **B** Representative photographs of isolated tumors on day 22. **C** Tumor growth kinetics across six treatment cohorts. **D** Weight of isolated tumors at day 22. **E** The body weight changes of nude mice in six groups. **F** Immunohistochemical (IHC) staining of AURKA, PTGS2, and Ki67 in tumor tissues, scale bar = 100 µm. **G**, **H** IHC scores of PTGS2 (**G)** and Ki67(**H**) in HCC tumor tissues. Data in (**D**) and (**G**, **H**) are presented as mean ± S.D. n = 5/group. Statistical significance was determined using two-way ANOVA (**C**, **E**) and one-way ANOVA with Tukey multiple comparisons test (**D**, **G**, **H**). (ns *P* ≥ 0.05, * *P* < 0.05, ** *P* < 0.01, *** *P* < 0.001, **** *P* < 0.0001).

Using sorafenib-resistant Huh7 (Huh7 SR) xenograft models, we next investigated whether combinating ferroptosis inducers with AURKA knockdown represents a viable therapeutic strategy for therapy-resistant tumors through ferroptosis induction in vivo (Fig. 3A). Since most ferroptosis inducers used in cell culture studies, including erastin and RSL3, are unsuitable for in vivo application, we employed imidazole ketone erastin (IKE), a potent erastin analog suitable for animal studies to induce ferroptosis [47]. Treatment with sorafenib or IKE alone showed limited efficacy in suppressing tumor growth. In striking contrast, AURKA knockdown, especially when combined with either agent, profoundly enhanced the therapeutic response, significantly reducing tumor growth rates and final tumor weights without adversely affecting body weight (Fig. 3B–E). Immunohistochemical (IHC) analysis further corroborated these findings, revealing that the combination regimes led to pronounced tumor suppression alongside hallmarks of enhanced ferroptotic activity—specifically, decreased Ki67 proliferation and elevated expression of the ferroptosis marker PTGS2 (Fig. 3F–H). Collectively, these in vivo results demonstrated that AURKA knockdown effectively sensitizes sorafenib-resistant HCC to ferroptosis. The marked antitumor effect achieved by co-targeting AURKA with ferroptosis induction highlights a promising therapeutic strategy to overcome acquired drug resistance in HCC.

### AURKA regulates ferroptosis resistance by suppressing ferritinophagy

**Fig. 4:**
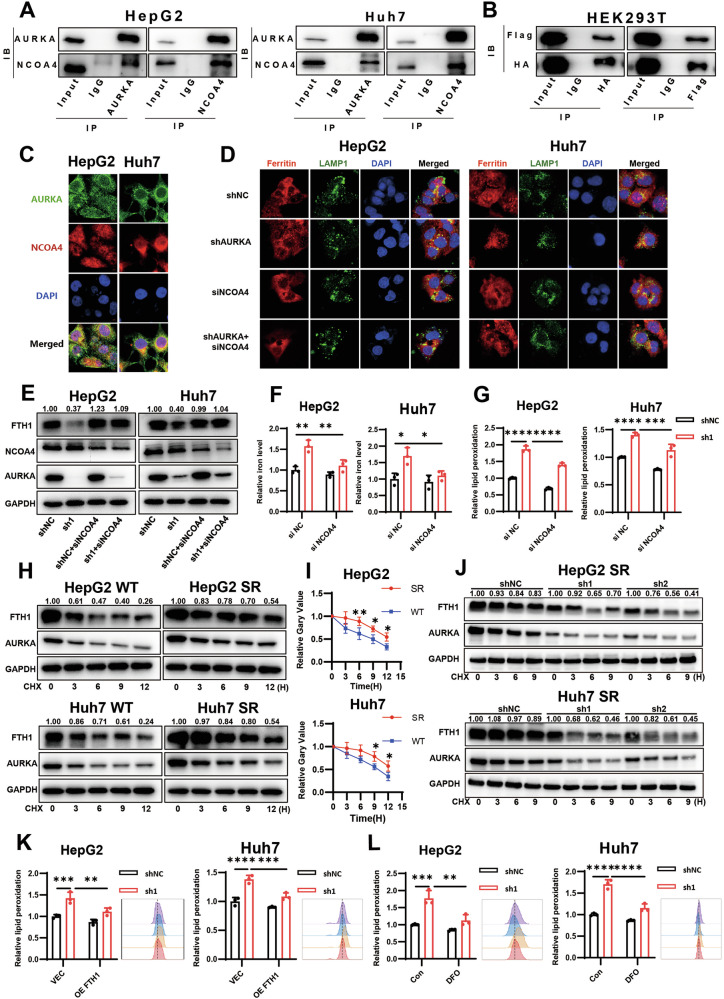
AURKA regulates ferroptosis resistance by suppressing ferritinophagy. **A** Co-immunoprecipitation (Co-IP) assay demonstrating endogenous NCOA4-FTH1 interaction in HepG2 and Huh7 cells. **B** Co-IP validation of exogenous HA-NCOA4 binding to Flag-AURKA and Myc-FTH1 in HEK293T cells. **C** Immunofluorescence (IF) showing co-localization of AURKA (green) and NCOA4 (red) in HCC cells, Scale bar: 5 µm. **D** Immunofluorescence detecting the co-localization of Ferritin (red) and the lysosomal marker LAMP1 (green), Scale bar: 5 µm. **E** Western blot analysis of FTH1 and NCOA4 protein expression levels in HCC cells with AURKA knockdown and/or NCOA4 silencing. **F** Detection of the intracellular iron levels in HCC cells with AURKA knockdown and/or NCOA4 silencing. **G** The lipid peroxidation levels in HCC cells with AURKA knockdown and/or NCOA4 silencing. **H, I** Cycloheximide (CHX, 20 μg/mL)-chase assay assessing FTH1 degradation kinetics in SR and WT cells. **J** Cycloheximide (CHX, 20 μg/mL)-chase assay assessing FTH1 degradation kinetics in SR cell lines after AURKA knockdown. **K** Lipid peroxidation levels in HCC cells with AURKA knockdown and/or FTH1 overexpression for 48 h**. L** Lipid peroxidation levels in AURKA-knockdown HCC cells exposed to deferoxamine (DFO, 40 μM) for 48 h. Data in (**F**, **G**, and **K**, **L**) are representative of three independent experiments and presented as mean ± S.D. Statistical significance was determined using one-way ANOVA with Tukey multiple comparisons test (**F**, **G, K**, **L**) and two-way ANOVA (**I**). (* *P* < 0.05, **P < 0.01, *** *P* < 0.001, **** *P* < 0.0001).

To further delineate the mechanism underlying AURKA-mediated ferroptosis resistance, we performed immunoprecipitation (IP) coupled with mass spectrometry (MS) to identify AURKA-interacting proteins. This approach identified over 2,000 putative AURKA interactors (Supplementary Table 1), including previously reported binding partners such as HNRNPK and NPM1, lending credibility to our MS results [48, 49]. KEGG pathway analysis revealed significant enrichment in the ferroptosis signaling pathway (Fig. S4A), corroborating AURKA’s regulatory role in ferroptosis. Notably, MS data highlighted a potential interaction between AURKA and NCOA4 (Nuclear Receptor Coactivator 4) (Fig. S4B), an autophagy receptor known to induce ferritinophagy by binding ferritin heavy chain (FTH1) and facilitating lysosomal iron release, thereby promoting ferroptosis [24]. Therefore, we proposed that AURKA may influence NCOA4-mediated ferritinophagy, thereby reducing the release of iron and inhibiting ferroptosis. Subsequently, we validated this interaction through reciprocal co-IP assays in HepG2 and Huh7 cells (Fig. 4A). Ectopic expression of Flag-tagged AURKA and HA-tagged NCOA4 in HEK293T cells also confirmed this direct binding (Fig. 4B). Additionally, immunofluorescence (IF) colocalization analysis showed that AURKA and NCOA4 colocalized in HCC cells. More importantly, the prominent co-localization signal was indeed primarily observed in the cytoplasmic compartment. This pattern is fully consistent with the proposed model wherein AURKA interacts with and regulates NCOA4 in the cytoplasm, where NCOA4 executes its key role in targeting ferritin for autophagic degradation (Fig. 4C and Fig. S4C).

To investigate whether AURKA regulates ferroptosis via NCOA4-mediated ferritinophagy, we first examined FTH1 degradation dynamics. AURKA knockdown significantly enhanced Ferritin-LAMP1 co-localization (Fig. 4D) and reduced FTH1 protein levels (Fig. 4E and Fig. S4D), indicating accelerated lysosomal degradation. This was accompanied by a marked rise in intracellular labile iron (Fig. 4F). Crucially, all these effects were abolished upon NCOA4 silencing (siNCOA4) (Fig. 4D–F), demonstrating that AURKA knockdown significantly promoted ferritinophagy in an NCOA4-mediated manner. Accordingly, the increase in lipid peroxidation and ROS induced by AURKA knockdown was attenuated when NCOA4 was depleted (Fig. 4G and Fig. S4E), confirming that AURKA suppresses ferroptosis by inhibiting NCOA4-driven ferritinophagy. We then asked whether this pathway is impaired in sorafenib-resistant cells. Compared with sensitive counterparts, SR cells exhibited delayed FTH1 degradation (Fig. 4H, I), suggesting suppressed ferritinophagy in resistant cells. Strikingly, AURKA knockdown in SR cells restored FTH1 degradation (Fig. 4J and Fig. S4F), implicating that AURKA stabilizes FTH1 protein to confer resistance. This regulation occurred post-transcriptionally, as FTH1 mRNA levels remained unchanged (Fig. S4G). Moreover, overexpression of FTH1 successfully reversed ferroptosis induced by AURKA knockdown (Fig. 4K and Fig. S4H–I), further supporting that AURKA regulates ferroptosis primarily through controlling FTH1 protein stability. The specificity of this axis was evidenced by unaltered expression of other iron-metabolism-associated proteins, TFRC1 and DMT1 (Fig. S4J–K).

To directly verify the role of ferrous ions, we treated cells with the iron chelator deferoxamine (DFO), which sequesters chelates intracellular free ferrous ions, and blocks Fenton reaction-driven ROS production, thereby inhibiting ferroptosis [12, 50]. Consistent with the changes observed upon NCOA4 depletion, DFO abrogated AURKA knockdown-induced ROS accumulation, lipid peroxidation, and growth inhibition (Fig. 4L and Fig. S4L–M). Together, these data collectively demonstrated that AURKA sustains ferroptosis resistance by interacting with NCOA4 to stabilize FTH1, thereby limiting free iron availability and suppressing Fenton reaction-driven oxidative stress.

### AURKA inhibits ferritinophagy by disrupting the NCOA4-FTH1 interaction in a kinase-dependent manner

**Fig. 5:**
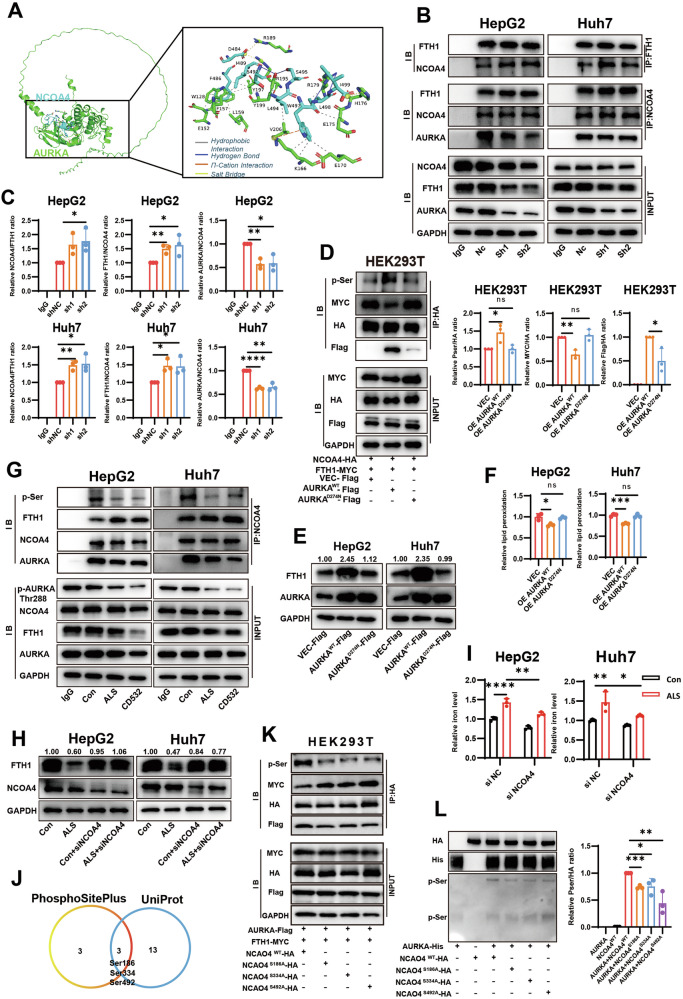
AURKA inhibits ferritinophagy by disrupting the NCOA4-FTH1 interaction in a kinase-dependent manner. **A** Molecular docking simulation of AURKA (green) binding to the NCOA4 C-terminal domain (aa 383-522, cyan). **B** Co-IP detection of the interactions between NCOA4 and AURKA or FTH1 following AURKA knockdown in HCC cells. **C** Quantification of NCOA4-FTH1 and AURKA-NCOA4 interactions upon AURKA knockdown in co-IP assays. **D** Co-IP and quantification analysis showing the phosphorylation of NCOA4 and interaction between NCOA4 and AURKA^WT^, AURKAD^274N^ or FTH1 in HEK293T cells. **E** Western blot validation of AURKA and FTH1 expression in HCC cells overexpressing AURKA^WT^ or AURKA^D274N^. **F** Lipid peroxidation levels in HCC cells overexpressing AURKA^WT^ or AURKA^D274N^. **G** Co-IP detection of the phosphorylation of NCOA4 and the interactions between NCOA4 and AURKA or FTH1 with ALS (1 μM) or CD532 (200 nM) treatment for 48 h. **H** Western blot analysis of FTH1 and NCOA4 expression in HCC cells treated with ALS (1 μM) or CD532 (200 nM) for 48 h. **I** Detection of the intracellular iron levels in HCC cells in the presence of ALS (1 μM) with or without NCOA4 depletion. **J** Venn diagram showing the predicted NCOA4 serine phosphorylation sites from UniProt and PhosphoSitePlus databases. **K** Co-IP detection of the serine phosphorylation and the interaction dynamics in HEK293T cells co-transfected with Myc-FTH1, Flag-AURKA, and HA-tagged NCOA4^WT^ or mutants (S186A/S234A/S492A). **L** Western blot and qualitative analysis of the serine phosphorylation of NCOA4 in *vitro* kinase assay. Data in (**C**, **D**, **F**, **I**, and **L**) are representative of three independent experiments and presented as mean ± S.D. Statistical significance was determined using one-way ANOVA with Tukey multiple comparisons test (**C**, **D**, **F**, **I**, **L**). (ns *P* ≥ 0.05, * *P* < 0.05, ** *P* < 0.01, *** *P* < 0.001, **** *P* < 0.0001).

Previous studies have reported that NCOA4 directly interacts with FTH1 via its C-terminal domain (aa 383-522) to mediate lysosome-dependent degradation of FTH1 and promote ferroptosis [51]. Given our finding that AURKA binds NCOA4 and suppresses NCOA4-mediated FTH1 degradation (Fig. 4A–E), we hypothesized that AURKA might competitively occupy specific domains of NCOA4 to inhibit ferroptosis. Molecular docking simulations between AURKA (UniProt ID: O14965) and NCOA4 (UniProt ID: Q13772) revealed multimodal binding forces—including hydrogen bonds and salt bridges—within the NCOA4 383-522 region (Fig. 5A), supporting a potential steric hindrance mechanism that could disrupt the NCOA4-FTH1 interaction.

To systematically validate this competitive binding mechanism, we performed sequential validation experiments. Endogenous co-immunoprecipitation assays demonstrated that AURKA knockdown enhanced NCOA4-FTH1 binding while weakening AURKA-NCOA4 association (Fig. 5B, C and Fig. S5A), indicating that AURKA competitively bound to NCOA4, thus disrupting NCOA4-FTH1 interaction and inhibiting ferritinophagy. Since AURKA is a serine/threonine kinase, we further investigated whether its catalytic activity was required. Wild-type AURKA (AURKA^WT^) strongly bound to NCOA4 and disrupted NCOA4-FTH1 interaction, whereas the kinase-deficient mutant AURKA^D274N^ showed reduced competitive binding capability (Fig. 5D). Phosphorylation-specific immunoblotting demonstrated that AURKA^WT^ significantly increased serine phosphorylation of NCOA4, an effect not observed with AURKA^D274N^ (Fig. 5D**)**. Functionally, AURKA^WT^ overexpression upregulated FTH1 protein levels (Fig. 5E and Fig. S5B) and suppressed ROS accumulation and lipid peroxidation (Fig. 5F and Fig. S5C), effects not observed with AURKA^D274N^. These results confirmed that AURKA-mediated suppression of ferritinophagy depends on its kinase activity.

We further probed this dependency using selective AURKA inhibitors, Alisertib (ALS) and CD532 [52, 53]. Similar to the effects of AURKA knockdown, pharmacological inhibition with ALS and CD532 reduced NCOA4 phosphorylation, weakened the AURKA-NCOA4 interaction, and enhanced NCOA4-FTH1 binding (Fig. 5G and Fig. S5D–G). These changes led to downregulated FTH1 protein levels and increased labile iron, effects rescued by NCOA4 knockdown (Fig. 5H–I and Fig. S5H–K). Consistent with restored ferroptotic susceptibility, ALS and CD532 induced concentration-dependent lipid peroxidation (Fig. S5L–M), while the iron chelator deferoxamine (DFO) rescued inhibitor-mediated proliferation suppression (Fig. S5N–O), further linking AURKA kinase activity to iron homeostasis.

To define the specific phosphorylation sites on NCOA4, we analyzed phosphoproteomic databases and identified three critical serine residues (Ser186/Ser234/Ser492) on NCOA4 (Fig. 5J). Alanine substitution mutants (S186A/S234A/S492A) significantly reduced serine phosphorylation, attenuated NCOA4-AURKA interaction, and restored NCOA4-FTH1 binding (Fig. 5K and Fig. S5P). In vitro kinase assays using purified proteins further confirmed that AURKA directly phosphorylated wild-type NCOA4, whereas phosphorylation of the triple mutant was nearly abolished (Fig. 5L and Fig. S5Q). This provides direct evidence that AURKA directly phosphorylates NCOA4 specifically at Ser186, Ser234, and Ser492.

In summary, these finding demonstrated that AURKA phosphorylates NCOA4 at Ser186/234/492 through its kinase activity, sterically hindering NCOA4-FTH1 interaction. This phosphorylation-dependent mechanism suppressed FTH1 degradation, limited labile Fe²⁺ release, and suppressed Fenton reaction-driven ferroptosis, thereby sustaining sorafenib resistance in HCC.

### Combination of AURKA inhibitors and ferroptosis inducers reverses acquired resistance in HCC

**Fig. 6:**
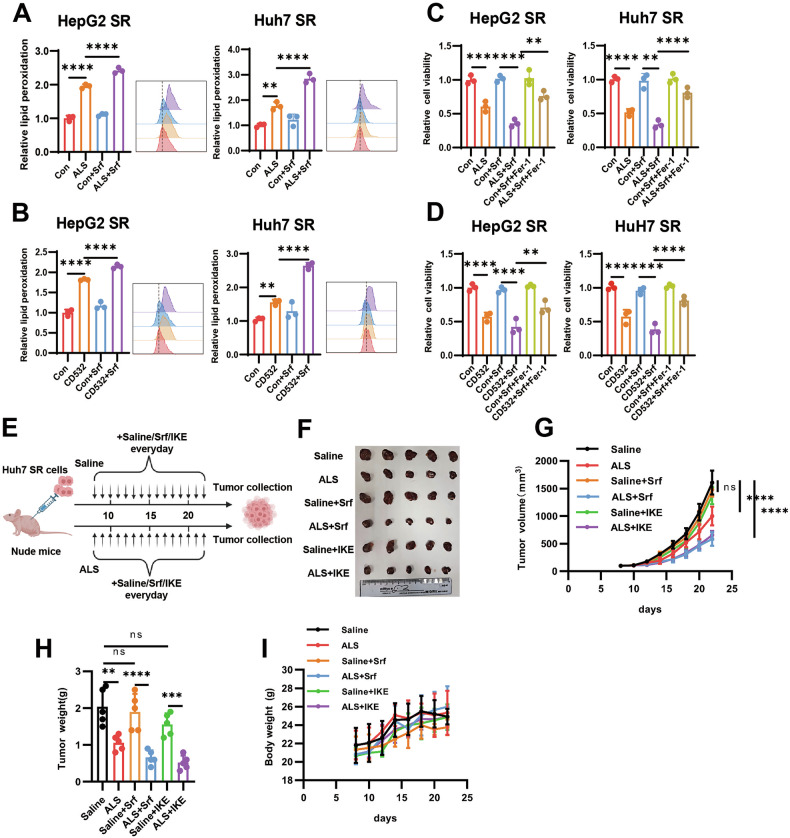
Combination of AURKA inhibitors and ferroptosis inducers reverses acquired resistance in HCC. **A**, **B** Lipid peroxidation levels in SR HCC cells treated with ALS (1 μM) (**A**) or CD532 (200 nM) (**B**) in the absence or presence of sorafenib (5 µM) for 48 h. **C**, **D** Cell viability of SR HCC cells treated with ALS (1 μM) (**C**) or CD532 (200 nM) (**D**) in the absence or presence of sorafenib (5 µM) or ferroptosis inhibitor Fer-1 (5 µM) for 48 h. Cell viability was assayed with CCK-8. **E** Schematic diagram of tumor inoculation and treatment in mice, arrows indicate the exact days for ALS, IKE, sorafenib, or saline administration. **F** Representative photographs of isolated tumors at day 22. **G** Tumor growth kinetics across six cohorts. **H** Weight of isolated tumors at day 22. **I** The body weight changes of nude mice in six groups. Data in (**A**–**D**) are representative of three independent experiments and presented as mean ± S.D. Data in (**H**) are representative of five tumors and presented as mean ± S.D. Statistical significance was determined using one-way ANOVA (**A–D**, **H**) and two-way ANOVA (**G**, **I**) with Tukey multiple comparisons test. (**P < 0.01, *** *P* < 0.001, **** *P* < 0.0001).

Given the kinase-dependent role of AURKA in suppressing ferritinophagy and regulating ferroptosis, we evaluated the therapeutic potential of combining selective AURKA inhibitors with ferroptosis inducers. Although sorafenib-resistant HCC cells exhibited strong resistance to ferroptosis induced by sorafenib monotherapy, co-treatment with AURKA inhibitors ALS or CD532 significantly and synergistically enhanced sorafenib-induced lipid peroxidation (Fig. 6A, B). Cell viability assays revealed that ALS and CD532 markedly increased the sensitivity of resistant cells to sorafenib, and these effects were reversed by the ferroptosis inhibitor Fer-1 (Fig. 6C, D). These data established that pharmacological AURKA inhibition restores ferroptotic susceptibility in therapy-resistant HCC.

This combinatorial strategy was further validated using the ferroptosis inducer RSL3. Both inhibitors potentiated RSL3-induced lipid peroxidation (Fig. S6A-B), and Fer-1 rescued the associated proliferation inhibition (Fig. S6C-D). The phenotypic concordance between pharmacological inhibition and genetic AURKA knockdown confirmed kinase activity-dependent regulation of ferroptosis by AURKA.

To assess translational potential, we established xenograft tumors using sorafenib-resistant Huh7 SR cells in nude mice (n = 5 per group). Tumor-bearing mice were stratified at day 8 post-implantation (when tumors reached approximately 100 mm^3^ tumors) into six cohorts: saline control, ALS monotherapy, sorafenib monotherapy, ALS combined with sorafenib, IKE monotherapy, and ALS combined with IKE (Fig. 6E). Consistent with the resistant phenotype, SR-derived tumors showed minimal response to sorafenib or IKE alone. In contrast, the combination of ALS with either sorafenib or IKE significantly suppressed tumor growth, as reflected in slower growth kinetics and lower final tumor weights (Fig. 6F–H). All regimens were well tolerated, with no significant changes in body weight observed across groups (Fig. 6I). Together, these in vivo data demonstrate that SR tumors remain refractory to single-agent sorafenib or ferroptosis induction, but are effectively re-sensitized by co-targeting AURKA. The marked synergy observed, without added toxicity, underscored the therapeutic potential of combining AURKA inhibition with ferroptosis inducers to overcome acquired resistance in HCC.

### The AURKA-FTH1 level serves as a predictor of unfavorable prognosis

**Fig. 7:**
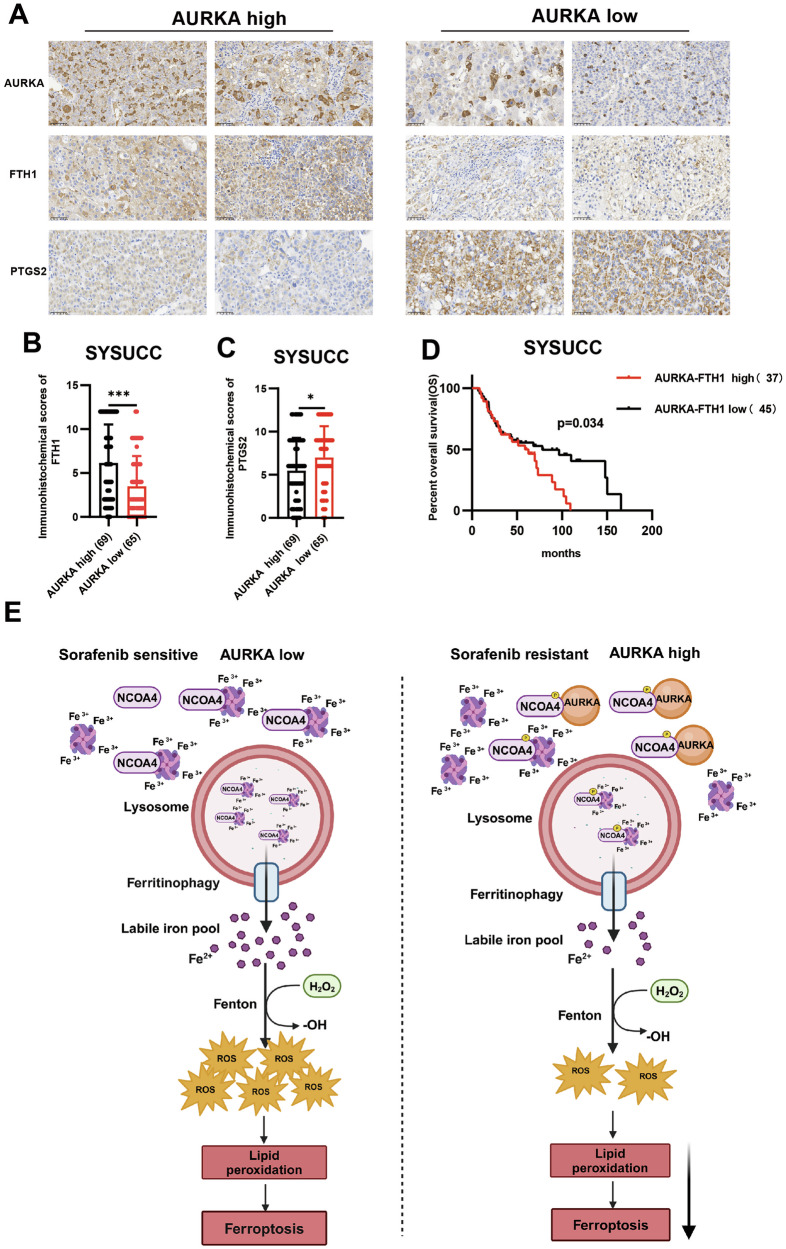
The AURKA-FTH1 level serves as a predictor of unfavorable prognosis. **A** Representative IHC staining of AURKA, FTH1, and PTGS2 in 134 HCC tumor tissues, scale bars = 50 μm. **B**, **C** Quantitative IHC scoring of FTH1 (**B**) and PTGS2 (**C**) expression in AURKA-high (n = 69) versus AURKA-low (n = 65) HCC cohorts. **D** Kaplan–Meier analysis of overall survival (OS) based on the AURKA-FTH1 expression level (n = 82). **E** Schematic model of AURKA in regulating ferroptosis in sorafenib-resistant HCC cells. Statistical analysis was performed by two-tailed Mann–Whitney test (**B**, **C**) and the Kaplan-Meier method with the log-rank test (**D**). (*P < 0.05, *** *P* < 0.001).

To evaluate the clinical relevance of AURKA and FTH1 in HCC, we conducted IHC analysis on a tissue microarray comprising 134 HCC specimens. Quantitative assessment revealed significantly elevated FTH1 protein expression in the AURKA-high cohort (n = 69) compared to the AURKA-low group (n = 65) (Fig. 7A, B), indicating a positive correlation between AURKA and FTH1 expression. Furthermore, the AURKA-high cohort exhibited substantially reduced expression of the ferroptosis biomarker PTGS2 (Fig. 7A, C), indicative of suppressed ferroptotic activity in tumors with elevated AURKA.

Finally, based on the Immunoreactivity Score (IRS), HCC patients were stratified into an AURKA-FTH1 high-expression cohort (n = 37) and a low-expression cohort (n = 45). Kaplan-Meier survival analysis demonstrated significantly shorter median overall survival (OS) in AURKA-FTH1 high-expression group (Fig. 7D), indicating that elevated AURKA-FTH1 expression was strongly associated with poor clinical prognosis in HCC. These clinical findings complemented our mechanistic data, positioning the AURKA-FTH1 regulatory axis as both a prognostic biomarker and therapeutic vulnerability in HCC. The inverse correlation between AURKA expression and ferroptosis activation further supported the clinical rationale for targeting this pathway to overcome therapy resistance.

## Discussion

Hepatocellular carcinoma (HCC) remains a critical clinical challenge due to its propensity for late-stage diagnosis and therapeutic resistance, with sorafenib resistance constituting a major barrier to improved outcomes [54–57]. Our study systematically investigates the mechanistic underpinnings of ferroptosis resistance in sorafenib-resistant HCC models, establishing the AURKA-NCOA4 regulatory axis as a novel determinant of therapeutic resistance through modulation of ferritinophagy. This work advances our understanding of ferroptosis dysregulation in HCC progression and identifies actionable targets for overcoming therapy resistance.

Using established sorafenib-resistant HCC cell lines (HepG2 SR and Huh7 SR), we confirmed a ferroptosis-resistant phenotype characterized by attenuated sorafenib-induced ROS accumulation and lipid peroxidation. Transcriptomic profiling revealed significant enrichment of ferroptosis-related pathways, including iron homeostasis and lipid metabolism, aligning with previous reports of ferroptosis change in therapy-resistant HCC. Notably, a previous study reported that sorafenib failed to induce ferroptosis in certain hepatoma cell lines [58]. This discrepancy likely stems from the context-dependent nature of ferroptosis susceptibility, which is governed by the cell-specific expression and activity of defensive components such as xCT, GPX4, and FSP1 [59, 60]. The variable responses to different ferroptosis inducers across cell lines, as also documented in the aforementioned study, further reinforce that ferroptotic outcomes are not universal but contingent upon the experimental model. Therefore, our work does not propose sorafenib as a universal ferroptosis inducer. Instead, our core finding is that within our specific parental and derived SR models, the cell death triggered by sorafenib and the acquired resistance are mechanistically linked to the ferroptosis pathway. This conclusion is robustly supported by the reversal of sorafenib-induced death in parental cells using the ferroptosis inhibitor ferrostatin-1 and, more critically, by the restoration of drug sensitivity in SR cells following disruption of the AURKA-NCOA4-FTH1 axis.

Within this framework, we identified AURKA as a central regulator of this resistant phenotype. Genetic ablation of AURKA not only restored sorafenib sensitivity in parental cells but also reversed ferroptosis resistance in refractory models, establishing AURKA as a critical negative regulator of ferroptotic cell death.

AURKA, a serine/threonine kinase and a classical oncogene, has been widely implicated in tumor progression by regulating cell proliferation, survival, and anti-apoptotic pathways [34, 61, 62]. Recent bioinformatics analyses have suggested AURKA as a potential ferroptosis regulator, but experimental validation has been lacking [63, 64]. In this study, we revealed that AURKA exerts its ferroptosis-suppressive effects through kinase-dependent modulation of ferritinophagy. Co-IP assays demonstrated that AURKA directly binds NCOA4 and phosphorylates three critical serine residues (S186/S334/S492), thereby competitively inhibiting NCOA4-FTH1 complex formation. This kinase-dependent disruption of ferritinophagy limits intracellular Fe²⁺ availability, effectively buffering against iron-catalyzed lipid peroxidation. Notably, this mechanism contrasts with a previous report showing that ATM-mediated phosphorylation at S550, which stabilizes NCOA4-FTH1 interactions to promote ferroptosis [65]. This discrepancy highlights the context-dependent regulation by distinct kinases in determining NCOA4 functional outcomes, underscoring the complexity of ferroptosis modulation through post-translational modifications.

Our findings provide a direct mechanistic explanation for the elevated FTH1 protein levels commonly observed in tumors. As the iron-storage protein, FTH1 sequesters labile ferrous ions, and its high expression is closely associated with ferroptosis suppression [66–68]. We demonstrated that AURKA knockdown significantly reduced FTH1 protein without affecting its mRNA, identifying a distinct, post-translational regulatory axis. While other studies have reported that high FTH1 expression can be transcriptionally regulated to inhibit ferroptosis [10, 69, 70]. and alterations in FTH1 mRNA may be indirectly linked to AURKA signaling or its downstream effectors [71]. Our work delineated a more immediate mechanism: AURKA directly stabilized FTH1 protein by phosphorylating NCOA4, thereby competitively inhibiting ferritinophagic degradation. This indicated that the established oncogenic function of AURKA contributes to tumorigenesis, at least in part, through the post-translational elevation of FTH1, which limits labile iron and suppresses ferroptosis. This adds a crucial functional layer to the understanding of FTH1 as an oncogene supporter—it serves not only as an iron-buffering unit but as a key survival factor, stabilized by AURKA to confer resistance to ferroptotic cell death.

Ferritinophagy is a process that degrades ferritin to release free iron ions, which plays a key role in modulating ferroptosis sensitivity [24]. As a core mediator of ferritinophagy, NCOA4 binds to FTH1 to facilitate autophagic degradation of ferritin, thereby releasing free ferrous iron and maintaining cellular iron homeostasis [23, 26]. Factors affecting the interaction between NCOA4 and FTH1 may influence ferritinophagy. A prior study reported that the benzimidazole derivative 9a directly binds to NCOA4, disrupting its interaction with FTH1 and inhibiting ferritinophagy [72]. Here, we demonstrate that AURKA directly binds to NCOA4 and competitively inhibits its interaction with FTH1, thereby blocking ferritinophagy. This supports the notion that targeting NCOA4-FTH1 binding promotes ferroptosis sensitivity. However, the dual role of ferritinophagy in tumor progression remains a subject of intense debate [73]. For example, in pancreatic cancer, NCOA4-mediated ferritinophagy is significantly upregulated, promoting Fe²⁺ release to fuel metabolic reprogramming and rapid tumor growth [30]. In acute myeloid leukemia (AML), ferritinophagy-derived Fe²⁺ is essential for leukemogenic self-renewal capacity [74]. In non-small cell lung cancer (NSCLC), adaptive upregulation of NCOA4-driven ferritinophagy maintains ron-sulfur cluster proteins in the electron transport chain and bolsters OXPHOS, thereby promoting therapy resistance [75]. Conversely, in many other malignancies, activation of ferritinophagy induces ferroptosis, suppressing tumor progression and enhancing treatment sensitivity [28, 76–78]. In this study, we demonstrate that AURKA/NCOA4 interaction inhibits ferritinophagy, reduces Fe²⁺ release, and confers ferroptosis resistance in sorafenib-resistant HCC, suggesting that enhanced ferritinophagy may paradoxically promote therapy sensitivity in this context. The dichotomy of ferritinophagy across cancers may stem from tumor-specific iron metabolic demands. Cancers like NSCLC and pancreatic cancer may rely on Fe²⁺-dependent energy metabolism, such as oxidation phosphorylation, to fuel growth. Threshold-dependent effects may also be at play: moderate ferritinophagy activation may sustain iron supply for proliferation, whereas excessive activation could induce lethal ROS/mitochondrial damage and ferroptosis. Additionally, tumor cells vary in their capacity to buffer ROS, leading to divergent ferroptosis thresholds upon ferritinophagy activation. These findings underscore the need for precise tailored therapeutic targeting of ferritinophagy, based on the iron metabolic profile and redox adaptability of specific tumor types.

The clinical relevance of our findings is reinforced by complementary studies. Gao et al. identified impaired FTH1 degradation in sorafenib-resistant HCC through liquid-liquid phase separation, while our work elucidates another FTH1 degradation suppression mechanism modulated by AURKA-NCOA4 interaction [79]. These parallel discoveries collectively implicate disrupted FTH1 autophagic degradation as a critical vulnerability in refractory HCC. Intriguingly, Ye et al. reported AURKA-mediated ferroptosis suppression in meningioma via Keap1/NRF2 signaling pathway, contrasting with our iron metabolism-centric mechanism, underscoring tissue-specific regulatory paradigms [71]. This divergence emphasizes the necessity for context-specific therapeutic strategies when targeting AURKA in combination with ferroptosis inducers.

However, several limitations of our study warrant consideration. First, our retrospective clinical analysis lacked detailed treatment response data, necessitating prospective validation in annotated HCC cohorts. Second, while phosphorylation at S186/S334/S492 was shown to disrupt NCOA4-FTH1 complex, the precise structural consequences require elucidation. Third, the generalizability of this mechanism to other sorafenib-resistant malignancies remains to be tested.

In summary, this study addresses the critical clinical challenge of sorafenib resistance in hepatocellular carcinoma by uncovering a novel, kinase-dependent mechanism through which AURKA directly suppresses ferritinophagy to drive ferroptosis resistance. We reveal, for the first time, that AURKA phosphorylates NCOA4 at specific serine residues, thereby competitively disrupting the NCOA4-FTH1 interaction and limiting intracellular labile iron—a direct biochemical link that extends beyond previously reported transcriptional regulatory networks. Our findings not only elucidate a precise molecular axis underlying acquired drug resistance but also provide strong preclinical validation: targeted inhibition of AURKA restores ferritinophagy and synergizes with ferroptosis inducers to overcome resistance in vivo. This work establishes the AURKA-NCOA4-FTH1 axis as a promising therapeutic target and proposes a translatable combination strategy, moving beyond phenotypic observation to offer a mechanistically grounded approach for overcoming therapy-resistant HCC.

## Supplementary information

Supplementary figures and figure legends

Supplementary Table 1

Original western blots

## Data Availability

The proteomics data generated in this study have been deposited to the ProteomeXchange Consortium via the iProX partner repository under accession number PXD075745. The mRNA sequences data have been deposited in the GEO under accession number GSE322742.

## References

[1] Bray F, Laversanne M, Sung H, Ferlay J, Siegel RL, Soerjomataram I, et al. Global cancer statistics 2022: GLOBOCAN estimates of incidence and mortality worldwide for 36 cancers in 185 countries. CA Cancer J Clin. 2024;74:229–263. 38572751 10.3322/caac.21834

[2] Marrero JA, Kulik LM, Sirlin CB, Zhu AX, Finn RS, Abecassis MM, et al. Diagnosis, staging, and management of hepatocellular carcinoma: 2018 practice guidance by the American Association for the study of liver diseases. Hepatology. 2018;68:723–750. 29624699 10.1002/hep.29913

[3] Xie D, Shi J, Zhou J, Fan J, Gao Q. Clinical practice guidelines and real-life practice in hepatocellular carcinoma: a Chinese perspective. Clin Mol Hepatol. 2022;29:206–216. 36545708 10.3350/cmh.2022.0402 PMC10121293

[4] Ren Z, Xu J, Bai Y, Xu A, Cang S, Du C, et al. Sintilimab plus a bevacizumab biosimilar (IBI305) versus sorafenib in unresectable hepatocellular carcinoma (ORIENT-32): a randomised, open-label, phase 2-3 study. Lancet Oncol. 2021;22:977–990. 34143971 10.1016/S1470-2045(21)00252-7

[5] Yau T, Park J-W, Finn RS, Cheng A-L, Mathurin P, Edeline J, et al. Nivolumab versus sorafenib in advanced hepatocellular carcinoma (CheckMate 459): a randomised, multicentre, open-label, phase 3 trial. Lancet Oncol. 2021;23:77–90. 34914889 10.1016/S1470-2045(21)00604-5

[6] Llovet JM, Ricci S, Mazzaferro V, Hilgard P, Gane E, Blanc J-F, et al. Sorafenib in advanced hepatocellular carcinoma. N Engl J Med. 2008;359:378–390. 18650514 10.1056/NEJMoa0708857

[7] Sankar K, Gong J, Osipov A, Miles SA, Kosari K, Nissen NN et al. Recent advances in the management of hepatocellular carcinoma. Clin Mol Hepatol. 2024;30;1-15. 10.3350/cmh.2023.0125 PMC10776289 37482076

[8] Huang C, Li Y, Zhang F, Zhang C, Ding Z. Advancements in elucidating the mechanisms of Sorafenib resistance in hepatocellular carcinoma. Int J Surg. 2025;111:2990–3005. 39992113 10.1097/JS9.0000000000002294 PMC12175829

[9] Xu J, Ji L, Ruan Y, Wan Z, Lin Z, Xia S, et al. UBQLN1 mediates sorafenib resistance through regulating mitochondrial biogenesis and ROS homeostasis by targeting PGC1β in hepatocellular carcinoma. Signal Transduct Target Ther. 2021;6:190. 34001851 10.1038/s41392-021-00594-4 PMC8129126

[10] Hao S-H, Ma X-D, Xu L, Xie J-D, Feng Z-H, Chen J-W, et al. Dual specific phosphatase 4 suppresses ferroptosis and enhances sorafenib resistance in hepatocellular carcinoma. Drug Resistance Updat. 2024;73:101052. 10.1016/j.drup.2024.101052 38262246

[11] Liao Y, Liu Y, Yu C, Lei Q, Cheng J, Kong W, et al. HSP90β impedes STUB1-induced ubiquitination of YTHDF2 to drive sorafenib resistance in hepatocellular carcinoma. Adv Sci (Weinh). 2023;10:e2302025. 37515378 10.1002/advs.202302025 PMC10520652

[12] Dixon SJ, Lemberg KM, Lamprecht MR, Skouta R, Zaitsev EM, Gleason CE, et al. Ferroptosis: an iron-dependent form of nonapoptotic cell death. Cell. 2012;149:1060–1072. 22632970 10.1016/j.cell.2012.03.042 PMC3367386

[13] Peng F, Liao M, Qin R, Zhu S, Peng C, Fu L, et al. Regulated cell death (RCD) in cancer: key pathways and targeted therapies. Signal Transduct Target Ther. 2022;7:286. 35963853 10.1038/s41392-022-01110-y PMC9376115

[14] Li J, Cao F, Yin H-L, Huang Z-J, Lin Z-T, Mao N, et al. Ferroptosis: past, present and future. Cell Death Dis. 2020;11:88. 32015325 10.1038/s41419-020-2298-2 PMC6997353

[15] Fu D, Wang C, Yu L, Yu R. Induction of ferroptosis by ATF3 elevation alleviates cisplatin resistance in gastric cancer by restraining Nrf2/Keap1/xCT signaling. Cell Mol Biol Lett. 2021;26:26. 34098867 10.1186/s11658-021-00271-y PMC8186082

[16] Jiang X, Stockwell BR, Conrad M. Ferroptosis: mechanisms, biology and role in disease. Nat Rev Mol Cell Biol. 2021;22:266–282. 33495651 10.1038/s41580-020-00324-8 PMC8142022

[17] Louandre C, Ezzoukhry Z, Godin C, Barbare J-C, Mazière J-C, Chauffert B, et al. Iron-dependent cell death of hepatocellular carcinoma cells exposed to sorafenib. Int J Cancer. 2013;133:1732–1742. 23505071 10.1002/ijc.28159

[18] Yao F, Deng Y, Zhao Y, Mei Y, Zhang Y, Liu X, et al. A targetable LIFR-NF-κB-LCN2 axis controls liver tumorigenesis and vulnerability to ferroptosis. Nat Commun. 2021;12:7333. 34921145 10.1038/s41467-021-27452-9 PMC8683481

[19] Yang C, Lu T, Liu M, Yuan X, Li D, Zhang J, et al. Tiliroside targets TBK1 to induce ferroptosis and sensitize hepatocellular carcinoma to sorafenib. Phytomedicine. 2023;111:154668. 36657316 10.1016/j.phymed.2023.154668

[20] Gao R, Kalathur RKR, Coto-Llerena M, Ercan C, Buechel D, Shuang S, et al. YAP/TAZ and ATF4 drive resistance to Sorafenib in hepatocellular carcinoma by preventing ferroptosis. EMBO Mol Med. 2021;13:e14351. 34664408 10.15252/emmm.202114351 PMC8649869

[21] Conrad M, Pratt DA. The chemical basis of ferroptosis. Nat Chem Biol. 2019;15:1137–1147. 31740834 10.1038/s41589-019-0408-1

[22] Zheng J, Conrad M. The metabolic underpinnings of ferroptosis. Cell Metab. 2020;32:920–937. 33217331 10.1016/j.cmet.2020.10.011

[23] Galy B, Conrad M, Muckenthaler M. Mechanisms controlling cellular and systemic iron homeostasis. Nat Rev Mol Cell Biol. 2023;25:133–155. 37783783 10.1038/s41580-023-00648-1

[24] Mancias JD, Wang X, Gygi SP, Harper JW, Kimmelman AC. Quantitative proteomics identifies NCOA4 as the cargo receptor mediating ferritinophagy. Nature. 2014;509:105–109. 24695223 10.1038/nature13148 PMC4180099

[25] Hou W, Xie Y, Song X, Sun X, Lotze MT, Zeh HJ, et al. Autophagy promotes ferroptosis by degradation of ferritin. Autophagy. 2016;12:1425–1428. 27245739 10.1080/15548627.2016.1187366 PMC4968231

[26] Gao M, Monian P, Pan Q, Zhang W, Xiang J, Jiang X. Ferroptosis is an autophagic cell death process. Cell Res. 2016;26:1021–1032. 27514700 10.1038/cr.2016.95 PMC5034113

[27] Dowdle WE, Nyfeler B, Nagel J, Elling RA, Liu S, Triantafellow E, et al. Selective VPS34 inhibitor blocks autophagy and uncovers a role for NCOA4 in ferritin degradation and iron homeostasis in vivo. Nat Cell Biol. 2014;16:1069–1079. 25327288 10.1038/ncb3053

[28] Li K, Chen B, Xu A, Shen J, Li K, Hao K, et al. TRIM7 modulates NCOA4-mediated ferritinophagy and ferroptosis in glioblastoma cells. Redox Biol. 2022;56:102451. 36067704 10.1016/j.redox.2022.102451 PMC9468590

[29] Huang G, Cai Y, Ren M, Zhang X, Fu Y, Cheng R, et al. Salidroside sensitizes Triple-negative breast cancer to ferroptosis by SCD1-mediated lipogenesis and NCOA4-mediated ferritinophagy. J Adv Res. 2024;74:589–607. 39353532 10.1016/j.jare.2024.09.027 PMC12302663

[30] Santana-Codina N, Del Rey MQ, Kapner KS, Zhang H, Gikandi A, Malcolm C, et al. NCOA4-mediated ferritinophagy is a pancreatic cancer dependency via maintenance of iron bioavailability for iron-sulfur cluster proteins. Cancer Discov. 2022;12:2180–2197. 35771492 10.1158/2159-8290.CD-22-0043 PMC9437572

[31] Nikonova AS, Astsaturov I, Serebriiskii IG, Dunbrack RL, Golemis EA. Aurora A kinase (AURKA) in normal and pathological cell division. Cell Mol Life Sci. 2012;70:661–687. 22864622 10.1007/s00018-012-1073-7 PMC3607959

[32] Grisetti L, Garcia CJC, Saponaro AA, Tiribelli C, Pascut D. The role of Aurora kinase A in hepatocellular carcinoma: unveiling the intriguing functions of a key but still underexplored factor in liver cancer. Cell Prolif. 2024;57:e13641. 38590119 10.1111/cpr.13641 PMC11294426

[33] Du R, Huang C, Liu K, Li X, Dong Z. Targeting AURKA in cancer: molecular mechanisms and opportunities for cancer therapy. Mol Cancer. 2021;20:15. 33451333 10.1186/s12943-020-01305-3 PMC7809767

[34] Yan M, Wang C, He B, Yang M, Tong M, Long Z, et al. Aurora-A kinase: a potent oncogene and target for cancer therapy. Med. Res. Rev. 2016;36:1036–1079. 27406026 10.1002/med.21399

[35] Manfredi MG, Ecsedy JA, Chakravarty A, Silverman L, Zhang M, Hoar KM, et al. Characterization of Alisertib (MLN8237), an investigational small-molecule inhibitor of aurora A kinase using novel in vivo pharmacodynamic assays. Clin Cancer Res. 2011;17:7614–7624. 22016509 10.1158/1078-0432.CCR-11-1536

[36] Görgün G, Calabrese E, Hideshima T, Ecsedy J, Perrone G, Mani M, et al. A novel Aurora-A kinase inhibitor MLN8237 induces cytotoxicity and cell-cycle arrest in multiple myeloma. Blood. 2010;115:5202–5213. 20382844 10.1182/blood-2009-12-259523 PMC2892955

[37] Friedberg JW, Mahadevan D, Cebula E, Persky D, Lossos I, Agarwal AB, et al. Phase II study of alisertib, a selective Aurora A kinase inhibitor, in relapsed and refractory aggressive B- and T-cell non-Hodgkin lymphomas. J Clin Oncol. 2013;32:44–50. 24043741 10.1200/JCO.2012.46.8793 PMC3867644

[38] O’Connor OA, Özcan M, Jacobsen ED, Roncero JM, Trotman J, Demeter J, et al. Randomized phase III study of alisertib or investigator’s choice (selected single agent) in patients with relapsed or refractory peripheral T-cell lymphoma. J Clin Oncol. 2019;37:613–623. 30707661 10.1200/JCO.18.00899 PMC6494247

[39] Falchook G, Coleman RL, Roszak A, Behbakht K, Matulonis U, Ray-Coquard I, et al. Alisertib in combination with weekly paclitaxel in patients with advanced breast cancer or recurrent ovarian cancer: a randomized clinical trial. JAMA Oncol. 2019;5:e183773. 30347019 10.1001/jamaoncol.2018.3773 PMC6439781

[40] Chen Y, Li Z, Cao Q, Guan H, Mao L, Zhao M. Ferroptosis-related gene signatures in neuroblastoma associated with prognosis. Front Cell Dev Biol. 2022;10:871512. 36147739 10.3389/fcell.2022.871512 PMC9486025

[41] Jia X, Tian J, Fu Y, Wang Y, Yang Y, Zhang M et al. Identification of AURKA as a Biomarker Associated with Cuproptosis and Ferroptosis in HNSCC. Int J Mol Sci. 2024;25;4372. 10.3390/ijms25084372 PMC11050640 38673957

[42] Luo L, Chen X, Huang F. Machine learning revealed ferroptosis features and ferroptosis-related gene-based immune microenvironment in lung adenocarcinoma. Chem Biol Interact. 2023;378:110471. 37061114 10.1016/j.cbi.2023.110471

[43] Kinoshita K, Noetzel TL, Pelletier L, Mechtler K, Drechsel DN, Schwager A, et al. Aurora A phosphorylation of TACC3/maskin is required for centrosome-dependent microtubule assembly in mitosis. J Cell Biol. 2005;170:1047–1055. 16172205 10.1083/jcb.200503023 PMC2171544

[44] Barros TP, Kinoshita K, Hyman AA, Raff JW. Aurora A activates D-TACC-Msps complexes exclusively at centrosomes to stabilize centrosomal microtubules. J Cell Biol. 2005;170:1039–1046. 16186253 10.1083/jcb.200504097 PMC2171528

[45] Otto T, Horn S, Brockmann M, Eilers U, Schüttrumpf L, Popov N, et al. Stabilization of N-Myc is a critical function of Aurora A in human neuroblastoma. Cancer Cell. 2009;15:67–78. 19111882 10.1016/j.ccr.2008.12.005

[46] Rio-Vilariño A, Cenigaonandia-Campillo A, García-Bautista A, Mateos-Gómez PA, Schlaepfer MI, Del Puerto-Nevado L, et al. Inhibition of the AURKA/YAP1 axis is a promising therapeutic option for overcoming cetuximab resistance in colorectal cancer stem cells. Br J Cancer. 2024;130:1402–1413. 38467828 10.1038/s41416-024-02649-z PMC11014903

[47] Zhang Y, Tan H, Daniels JD, Zandkarimi F, Liu H, Brown LM et al. Imidazole ketone erastin induces ferroptosis and slows tumor growth in a mouse lymphoma model. Cell Chem Biol. 2019;26;623-633. 10.1016/j.chembiol.2019.01.008 PMC6525071 30799221

[48] Chen H, Hu J, Xiong X, Chen H, Lin B, Chen Y, et al. AURKA inhibition induces Ewing’s sarcoma apoptosis and ferroptosis through NPM1/YAP1 axis. Cell Death Dis. 2024;15:99. 38287009 10.1038/s41419-024-06485-0 PMC10825207

[49] Li S, Qi Y, Yu J, Hao Y, Xu L, Ding X, et al. Aurora kinase A regulates cancer-associated RNA aberrant splicing in breast cancer. Heliyon. 2023;9:e17386. 37415951 10.1016/j.heliyon.2023.e17386 PMC10320321

[50] Lei L, Yuan J, Dai Z, Xiang S, Tu Q, Cui X, et al. Targeting the Labile Iron Pool with Engineered DFO Nanosheets to Inhibit Ferroptosis for Parkinson’s Disease Therapy. Adv Mater. 2024;36:e2409329. 39221531 10.1002/adma.202409329

[51] Mancias JD, Pontano Vaites L, Nissim S, Biancur DE, Kim AJ, Wang X et al. Ferritinophagy via NCOA4 is required for erythropoiesis and is regulated by iron dependent HERC2-mediated proteolysis. Elife. 2015;4;e10308. 10.7554/eLife.10308 PMC4592949 26436293

[52] Gilburt JAH, Sarkar H, Sheldrake P, Blagg J, Ying L, Dodson CA. Dynamic Equilibrium of the AuroraA Kinase Activation Loop Revealed by Single-Molecule Spectroscopy. Angew Chem Int Ed Engl. 2017;56:11409–11414. 28700101 10.1002/anie.201704654 PMC5601181

[53] Musavizadeh Z, Grottesi A, Guarguaglini G, Paiardini A. Phosphorylation, Mg-ADP, and Inhibitors Differentially Shape the Conformational Dynamics of the A-Loop of Aurora-A. Biomolecules. 2021;11;567. 10.3390/biom11040567 PMC8070005 33921540

[54] Marrero JA, Kulik LM, Sirlin CB, Zhu AX, Finn RS, Abecassis MM, et al. Diagnosis, Staging, and Management of Hepatocellular Carcinoma: 2018 Practice Guidance by the American Association for the Study of Liver Diseases. Hepatol (Baltim, Md). 2018;68:723–750. 10.1002/hep.29913 29624699

[55] Cheng Z, Wei-Qi J, Jin D. New insights on sorafenib resistance in liver cancer with correlation of individualized therapy. Biochim Biophys Acta Rev Cancer. 2020;1874:188382. 32522600 10.1016/j.bbcan.2020.188382

[56] Xia S, Pan Y, Liang Y, Xu J, Cai X. The microenvironmental and metabolic aspects of sorafenib resistance in hepatocellular carcinoma. EBioMedicine. 2020;51:102610. 31918403 10.1016/j.ebiom.2019.102610 PMC7000339

[57] Zhang W, Hong X, Xiao Y, Wang H, Zeng X. Sorafenib resistance and therapeutic strategies in hepatocellular carcinoma. Biochim Biophys Acta Rev Cancer. 2025;1880:189310. 40187502 10.1016/j.bbcan.2025.189310

[58] Zheng J, Sato M, Mishima E, Sato H, Proneth B, Conrad M. Sorafenib fails to trigger ferroptosis across a wide range of cancer cell lines. Cell Death Dis. 2021;12:698. 34257282 10.1038/s41419-021-03998-w PMC8277867

[59] Dixon SJ, Olzmann JA. The cell biology of ferroptosis. Nat Rev Mol Cell Biol. 2024;25:424–442. 38366038 10.1038/s41580-024-00703-5 PMC12187608

[60] Lee WC, Dixon SJ. Mechanisms of ferroptosis sensitization and resistance. Dev Cell. 2025;60:982–993. 40199240 10.1016/j.devcel.2025.02.004 PMC12883161

[61] Chen C, Song G, Xiang J, Zhang H, Zhao S, Zhan Y. AURKA promotes cancer metastasis by regulating epithelial-mesenchymal transition and cancer stem cell properties in hepatocellular carcinoma. Biochem Biophys Res Commun. 2017;486:514–520. 28322787 10.1016/j.bbrc.2017.03.075

[62] Chen M, Zhu H, Li J, Luo D, Zhang J, Liu W, et al. Research progress on the relationship between AURKA and tumorigenesis: the neglected nuclear function of AURKA. Ann Med. 2024;56:2282184. 38738386 10.1080/07853890.2023.2282184 PMC11095293

[63] Hu J, Song F, Kang W, Xia F, Song Z, Wang Y, et al. Integrative analysis of multi-omics data for discovery of ferroptosis-related gene signature predicting immune activity in neuroblastoma. Front Pharm. 2023;14:1162563. 10.3389/fphar.2023.1162563 PMC10373597 37521469

[64] Chung TT, Piao Z, Lee SJ. Identification of ferroptosis-related signature predicting prognosis and therapeutic responses in pancreatic cancer. Sci Rep. 2025;15:75. 39748113 10.1038/s41598-024-84607-6 PMC11695983

[65] Wu H, Liu Q, Shan X, Gao W, Chen Q. ATM orchestrates ferritinophagy and ferroptosis by phosphorylating NCOA4. Autophagy. 2023;19:2062–2077. 36752571 10.1080/15548627.2023.2170960 PMC10283418

[66] Kong N, Chen X, Feng J, Duan T, Liu S, Sun X, et al. Baicalin induces ferroptosis in bladder cancer cells by downregulating FTH1. Acta Pharm Sin B. 2021;11:4045–4054. 35024325 10.1016/j.apsb.2021.03.036 PMC8727776

[67] Liu Y, Sun Q, Guo J, Yan L, Yan Y, Gong Y, et al. Dual ferroptosis induction in N2-TANs and TNBC cells via FTH1 targeting: A therapeutic strategy for triple-negative breast cancer. Cell Rep Med. 2025;6:101915. 39809268 10.1016/j.xcrm.2024.101915 PMC11866498

[68] Duan Z-W, Wang W-T, Wang Y, Wang R, Hua W, Shang C-Y, et al. SH3GL1-activated FTH1 inhibits ferroptosis and confers doxorubicin resistance in diffuse large B-cell lymphoma. Clin Transl Med. 2025;15:e70246. 40038872 10.1002/ctm2.70246 PMC11879899

[69] Zhang X-Y, Li S-S, Gu Y-R, Xiao L-X, Ma X-Y, Chen X-R, et al. CircPIAS1 promotes hepatocellular carcinoma progression by inhibiting ferroptosis via the miR-455-3p/NUPR1/FTH1 axis. Mol Cancer. 2024;23:113. 38802795 10.1186/s12943-024-02030-x PMC11131253

[70] Zhang R, Pan T, Xiang Y, Zhang M, Xie H, Liang Z, et al. Curcumenol triggered ferroptosis in lung cancer cells via lncRNA H19/miR-19b-3p/FTH1 axis. Bioact Mater. 2021;13:23–36. 35224289 10.1016/j.bioactmat.2021.11.013 PMC8843976

[71] Ye Y, Xu L, Zhang L, Zhao P, Cai W, Fu G, et al. Meningioma achieves malignancy and erastin-induced ferroptosis resistance through FOXM1-AURKA-NRF2 axis. Redox Biol. 2024;72:103137. 38642502 10.1016/j.redox.2024.103137 PMC11047291

[72] Fang Y, Chen X, Tan Q, Zhou H, Xu J, Gu Q. Inhibiting Ferroptosis through Disrupting the NCOA4-FTH1 Interaction: A New Mechanism of Action. ACS Cent Sci. 2021;7:980–989. 34235259 10.1021/acscentsci.0c01592 PMC8227600

[73] Li J-Y, Feng Y-H, Li Y-X, He P-Y, Zhou Q-Y, Tian Y-P, et al. Ferritinophagy: A novel insight into the double-edged sword in ferritinophagy-ferroptosis axis and human diseases. Cell Prolif. 2024;57:e13621. 38389491 10.1111/cpr.13621 PMC11216947

[74] Larrue C, Mouche S, Angelino P, Sajot M, Birsen R, Kosmider O, et al. Targeting ferritinophagy impairs quiescent cancer stem cells in acute myeloid leukemia in vitro and in vivo models. Sci Transl Med. 2024;16:eadk1731. 39047119 10.1126/scitranslmed.adk1731

[75] Wang H, Hu Q, Chen Y, Huang X, Feng Y, Shi Y, et al. Ferritinophagy mediates adaptive resistance to EGFR tyrosine kinase inhibitors in non-small cell lung cancer. Nat Commun. 2024;15:4195. 38760351 10.1038/s41467-024-48433-8 PMC11101634

[76] Qin X, Zhang J, Wang B, Xu G, Yang X, Zou Z, et al. Ferritinophagy is involved in the zinc oxide nanoparticles-induced ferroptosis of vascular endothelial cells. Autophagy. 2021;17:4266–4285. 33843441 10.1080/15548627.2021.1911016 PMC8726675

[77] Wang J, Wu N, Peng M, Oyang L, Jiang X, Peng Q, et al. Ferritinophagy: research advance and clinical significance in cancers. Cell Death Discov. 2023;9:463. 38110359 10.1038/s41420-023-01753-y PMC10728094

[78] Wang X, Xu L, Meng Y, Chen F, Zhuang J, Wang M, et al. FOXO1-NCOA4 Axis Contributes to Cisplatin-Induced Cochlea Spiral Ganglion Neuron Ferroptosis via Ferritinophagy. Adv Sci (Weinh). 2024;11:e2402671. 39206719 10.1002/advs.202402671 PMC11515924

[79] Gao Y, Tong M, Wong T-L, Ng K-Y, Xie Y-N, Wang Z, et al. Long noncoding RNA URB1-antisense RNA 1 (AS1) suppresses sorafenib-induced ferroptosis in hepatocellular carcinoma by driving ferritin phase separation. ACS Nano. 2023;17:22240–22258. 37966480 10.1021/acsnano.3c01199

