# Supplementary material for: AURKA suppresses NCOA4-mediated ferritinophagy to enhance sorafenib resistance in hepatocellular carcinoma
Source: Cell Death Dis. 2026 Apr 24;17(1):540. doi: 10.1038/s41419-026-08774-2 (PMC13237277; doi:10.1038/s41419-026-08774-2)
Supplement: Supplementary file 1 — Supplementary figures and figure legends [file 41419_2026_8774_MOESM1_ESM.doc]

**
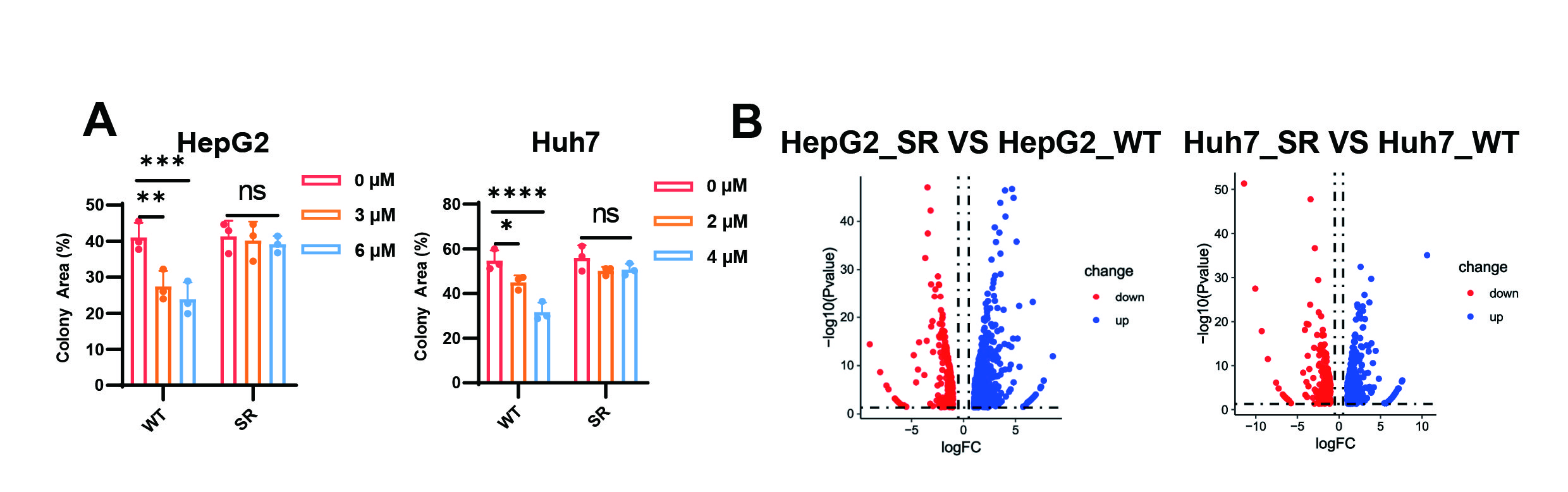
**

**Fig. S1 (Extended data related to Fig 1)**

**A** Quantitative analysis of the colony formation assay. **B** Volcano plots of differentially expressed genes (DEGs, |log2FC| >0.5, p <0.05) from RNA sequencing in GSE121153 dataset. Data in **A** are representative of three independent experiments and presented as mean ± S.D. Statistical analysis was performed by one-way ANOVA with Tukey multiple comparisons test (**A**).(ns *P* ≥ 0.05, **P*< 0.05, ****P*< 0.001)

**
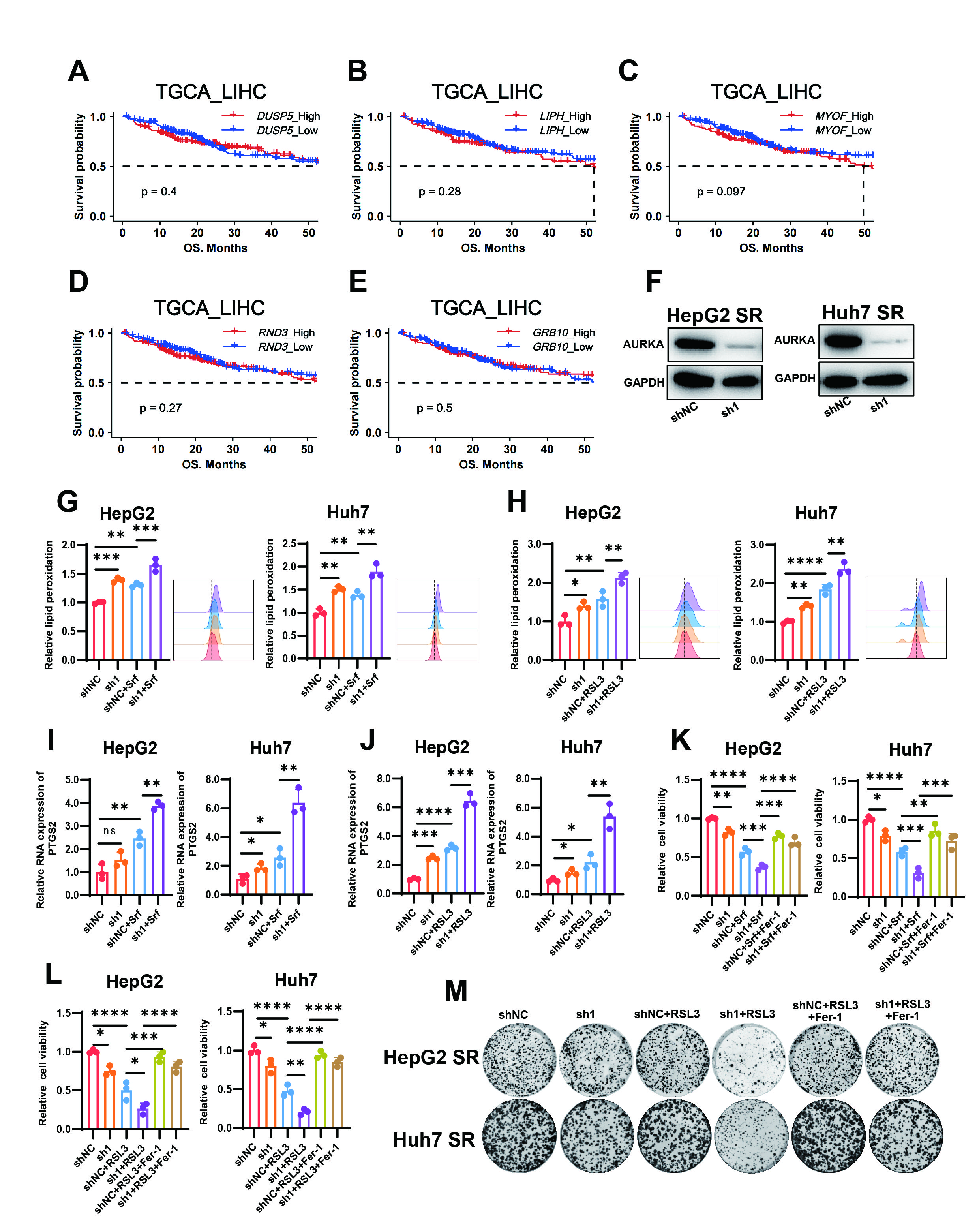
**

**Fig. S2 (Extended data related to Fig 2)**

1. **E** Kaplan-Meier survival analysis of overall survival (OS) in HCC patients from the TCGA cohort stratified by mRNA expression levels of *DUSP5* (**A)**, *LIPH* **（B）,** *MYOF* **(C),** *RND3* **(D)** and *GRB10* (**E)**. **F** AURKA knockdown validation in SR HCC cells transduced with Lv-shAURKA vs Lv-NC. **G-H** Detection of the lipid peroxidation levels with BODIPY-C11 probe in AURKA-knockdown WT HCC cells treated with Sorafenib **(G)** or RSL3 **(H)**. **I-J** PTGS2 mRNA expression by qRT-PCR in AURKA-knockdown HepG2 and Huh7 cells treated with Sorafenib **(I)** or RSL3 **(J)**. **K-L** CCK-8 assay showing cell viability in AURKA-knockdown WT HCC cells in the absence or presence ofSorafenib **(K)** or RSL3 **(L)** or ferroptosis inhibitor Fer-1 (5 µM). (HepG2:0.5 μM; Huh7:100 nM) for 48 h. **M** Colony formation assay of AURKA-knockdown SR HCC cells in the absence or presence of RLS or ferroptosis inhibitor Fer-1 (5 µM). Data in **G-L** are representative of three independent experiments and presented as mean ± S.D. Statistical analysis was performed by the Kaplan-Meier method with the log-rank test (**A-E**).and one-way ANOVA with Tukey multiple comparisons test (**G-L**). (ns *P* ≥ 0.05, **P*< 0.05, ***P*< 0.01, ****P*< 0.001, *****P*< 0.0001)


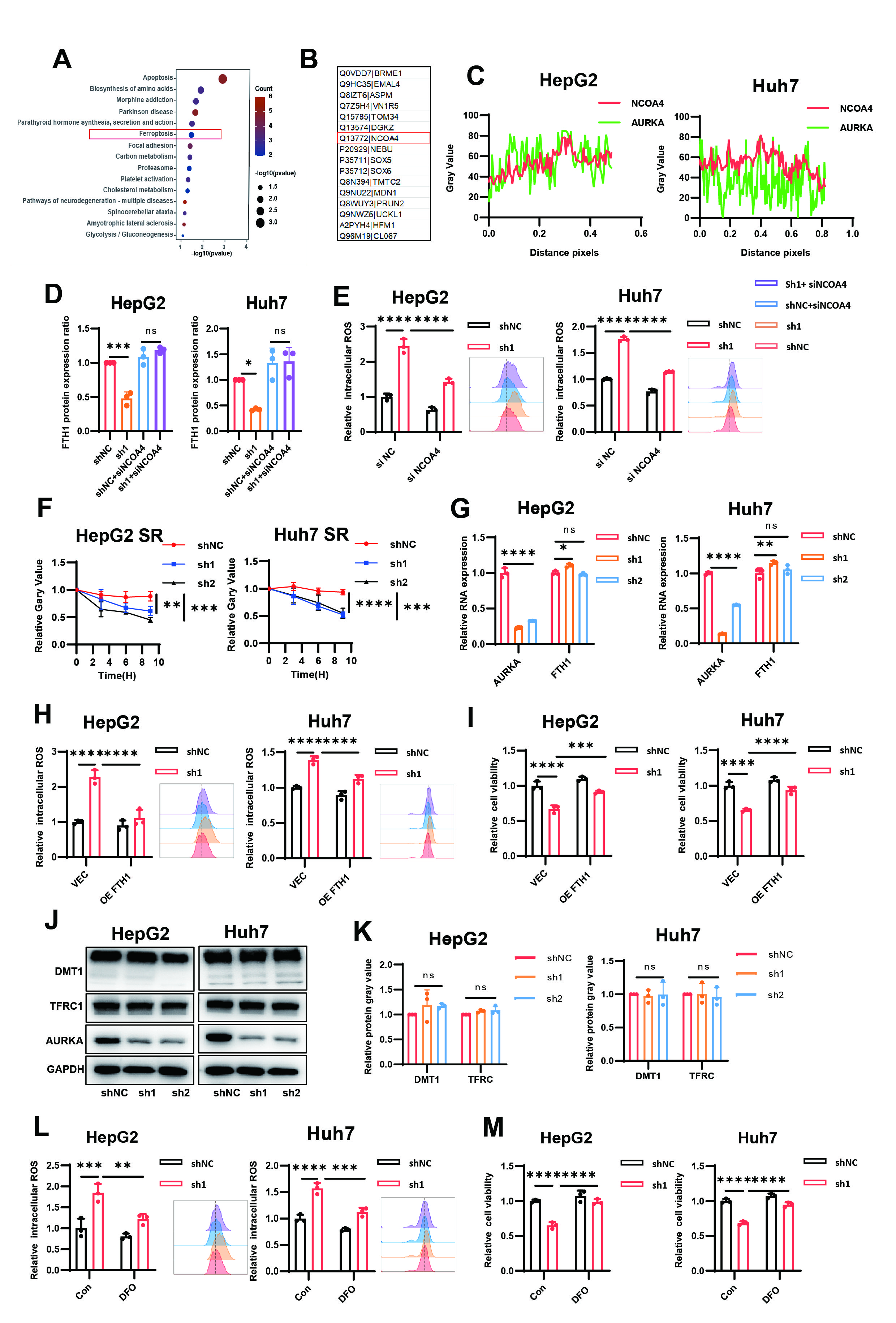


**Fig. S4 (Extended data related to Fig 4)**

**A** KEGG pathway enrichment analysis of AURKA-interacting proteins identified by mass spectrometry (MS). **B** Partial display of proteomic MS results binding with AURKA.**C** Quantitative immunofluorescence co-localization analysis of AURKA (green) and NCOA4 (red). **D** Quantification of FTH1 protein expression by densitometry. **E** ROS levels in AURKA-knockdown HCC cells with or without NCOA4 silencing. **F** Quantification of FTH1 degradation kinetics (cycloheximide chase assay) in SR cells after AURKA knockdown. **G** FTH1 mRNA levels by qRT-PCR in AURKA-knockdown HCC cells. **H** ROS levels in AURKA-knockdown HCC cells with or without FTH1 overexpression for 48 h. **I** Cell viability of AURKA-knockdown HCC cells with or without FTH1 overexpression. **J** Western blot analysis of TFRC1 and DMT1 expression in AURKA-knockdown HCC cells. **K** Quantification of TFRC1 and DMT1 protein expression by densitometry. **L-M** Intracellular ROS levels **(L)** and cell viability **(M)** in AURKA-knockdown HCC cells in the absence or presence of deferoxamine (DFO, 40 µM) for 48 h. Data represent as mean ± SD. Data in **D-E**, **G-I and K-M** are representative of three independent experiments and presented as mean ± S.D. Statistical analysis was performed by one-way ANOVA with Tukey multiple comparisons test.(**D-E, G-I, K-M**) and two-way ANOVA (**F**). (ns *P* ≥ 0.05, **P*< 0.05, ***P*< 0.01, ****P*< 0.001, *****P*< 0.0001)


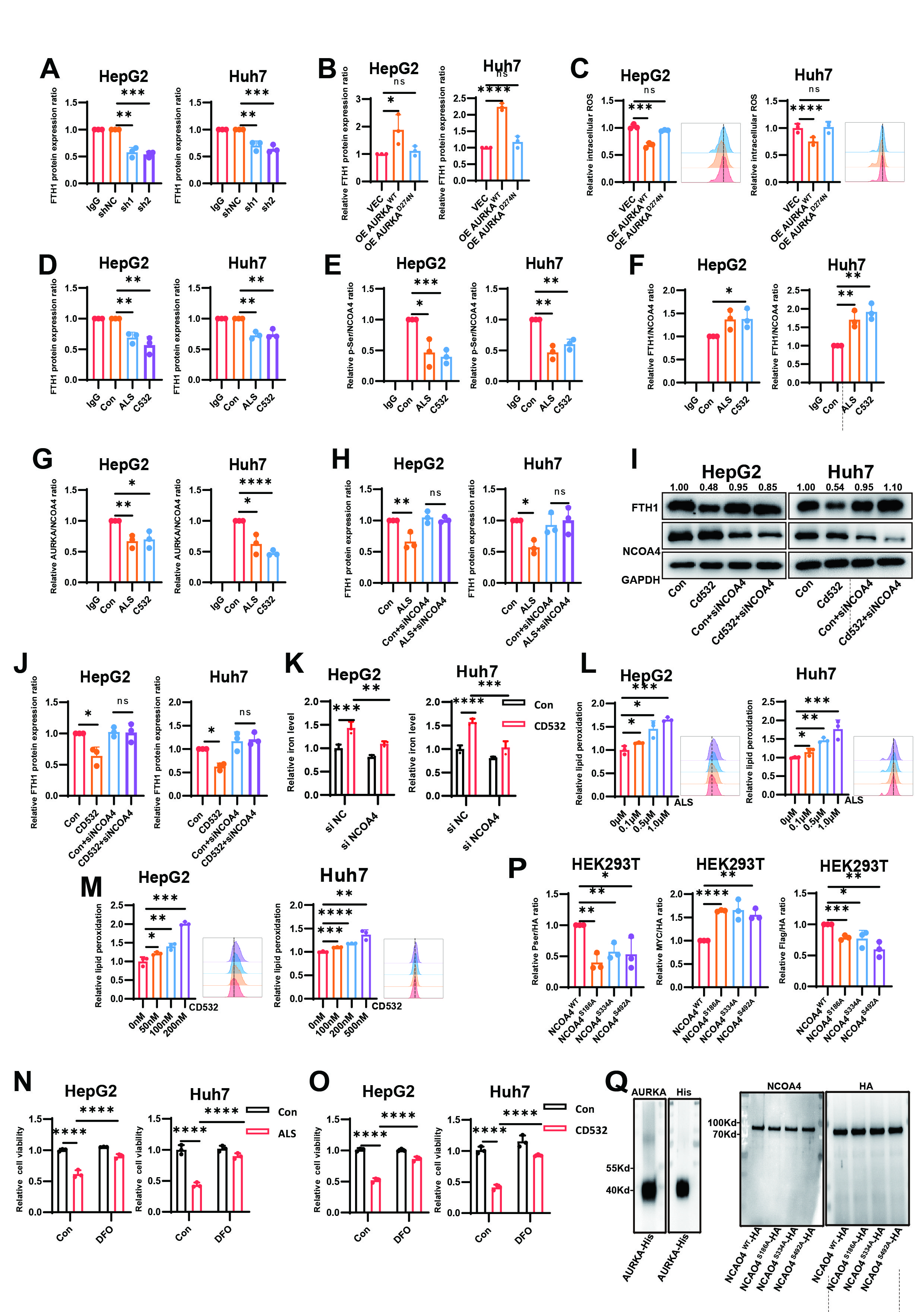


**Fig. S5 (Extended data related to Fig 5)**

**A** Quantitative analysis of FTH1 in INPUT samples from the co-IP assay upon AURKA konckdown. **B** Quantitative analysis of FTH1 in HCC cells overexpressing AURKA^WT^ or AURKAD^274N^. **C** Intracellular ROS levels in HCC cells overexpressing wild-type AURKA (AURKA^WT^) or kinase-dead mutant AURKA (AURKA^D274N^). **D** Quantitative analysis of FTH1 in the INPUT samples from the co-IP assay treated with ALS or CD532. **E-G** Quantitative analysis of NCOA4 phosphorylation **(E)**, NCOA4-FTH1 binding **(F)** and NCOA4-AURKA binding **(G)** ability in IP samples from the co-IP assay treated with ALS or CD532. **H** Quantitative analysis of FTH1 expression treated with ALS (1 µM, 48 h) with or without NCOA4 depletion. **I-J** Western blot and quantitative analysis of FTH1 expression treated with CD532 (200 nM, 48 h) with or without NCOA4 depletion. **K** Intracellular iron levels in HCC cells treated with CD532 (200 nM, 48 h) with or without NCOA4 depletion. **L-M** Lipid peroxidation levels in HCC cells treated with ALS (1 µM) **(L)** or CD532 (200 nM) **(M)** for 48 h. **N-O** Cell viability of HCC cells treated with with ALS (1 µM) **(N)** or CD532 (200 nM) **(O)** in the absence or presence of deferoxamine (DFO,40 µM) for 48 h. **P** Quantitative analysis of phosphorylation of NCOA4, NCOA4-FTH1 binding and NCOA4-AURKA binding in IP samples from the co-IP assay. **Q** Western blot verifying the purity and molecular weight of the recombinant proteins. Data in **A-H** and **J-P** are representative of three independent experiments and presented as mean ± S.D. Statistical analysis was performed by one-way ANOVA with Tukey multiple comparisons test.(**A-H, J-P**). (ns *P* ≥ 0.05, **P*< 0.05, ***P*< 0.01, ****P*< 0.001, *****P*< 0.0001)


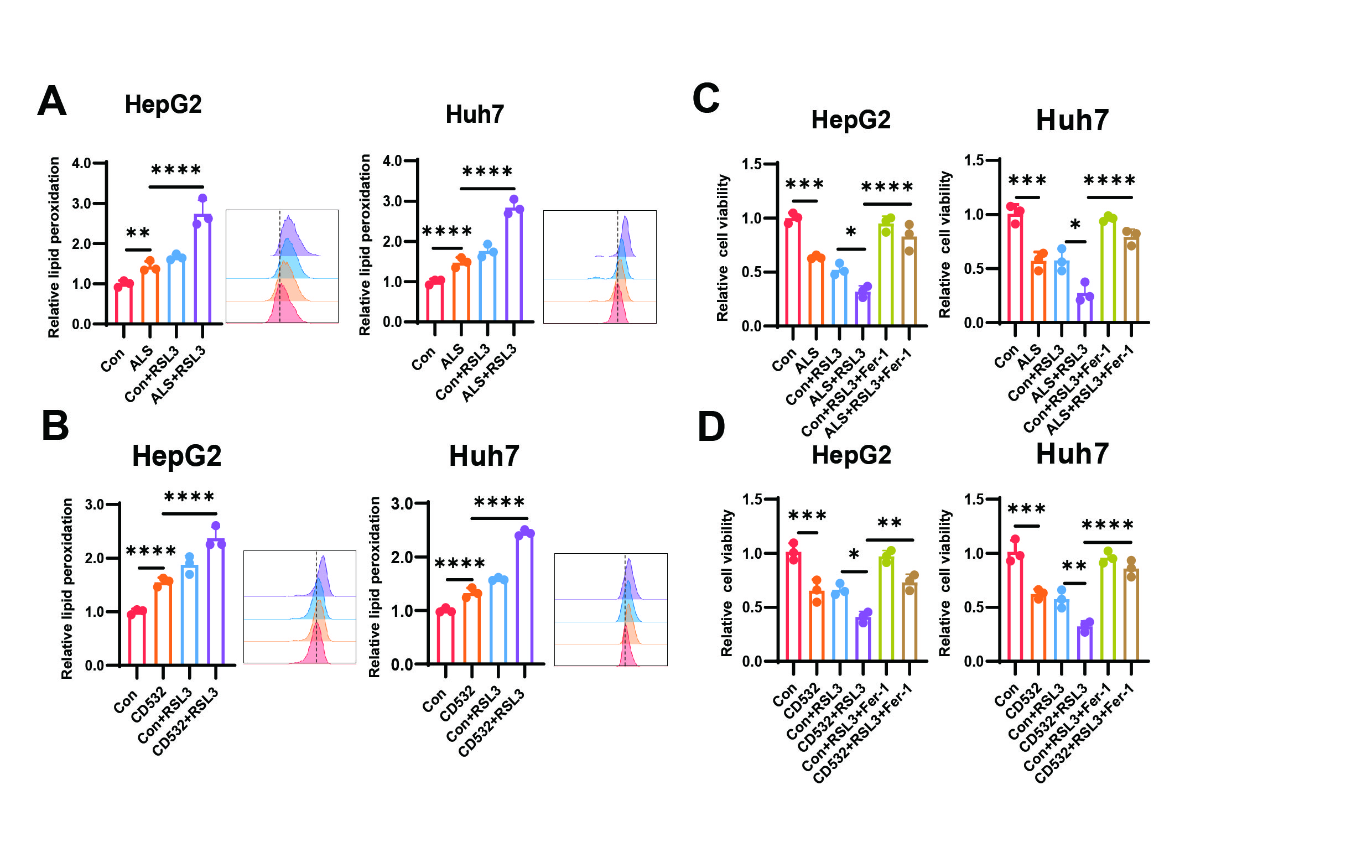
**Fig. S6 (Extended data related to Fig 6)**

**A-B** Lipid peroxidation levels in HCC cells treated with ALS (1 µM) **(A)** or CD532 (200 nM) **(B)** in the absence or presence of RSL3 (HepG2: 0.5 µM; Huh7: 100 nM) for 48 h. **C-D** Cell viability of HCC cells treated with ALS (1 µM) **(C)** or CD532 (200 nM) **(D)** in the absence or presence of RSL3 (HepG2: 0.5 µM; Huh7: 100 nM) for 48 h. Data in **A-D** are representative of three independent experiments and presented as mean ± S.D. Statistical analysis was performed by one-way ANOVA with Tukey multiple comparisons test (**A-D**).(**P*< 0.05, ***P*< 0.01, ****P*< 0.001, *****P*< 0.0001)
