# Supplementary material for: AURKA suppresses NCOA4-mediated ferritinophagy to enhance sorafenib resistance in hepatocellular carcinoma
Source: Cell Death Dis. 2026 Apr 24;17(1):540. doi: 10.1038/s41419-026-08774-2 (PMC13237277; doi:10.1038/s41419-026-08774-2)

Fig.2E

Fig2E.AURKA-HepG2(left panel)
Huh7(right panel)

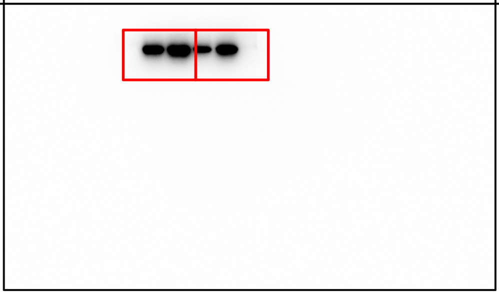

Fig2E.GAPDH-HepG2(left panel)
Huh7(right panel)

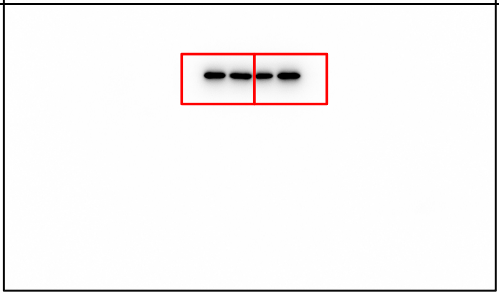

Fig.2F

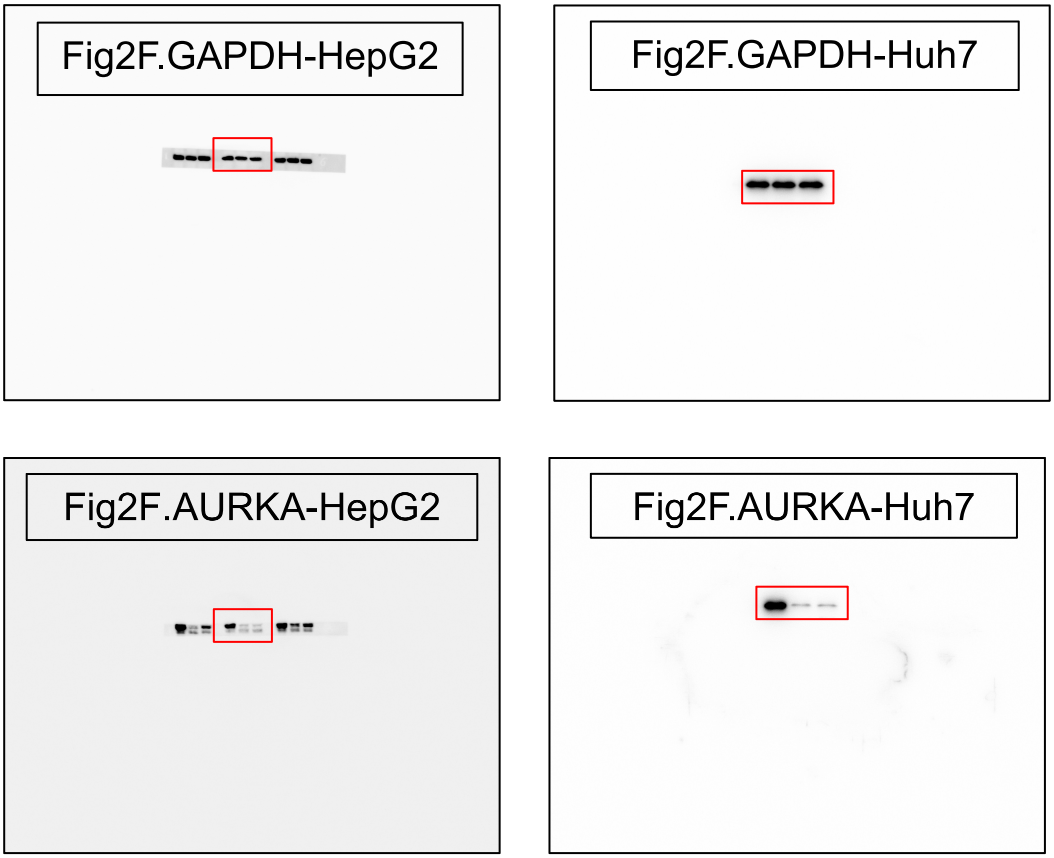
Fig2F.GAPDH-HepG2
Fig2F.GAPDH-Huh7
Fig2F.AURKA-HepG2
Fig2F.AURKA-Huh7

Fig.4A

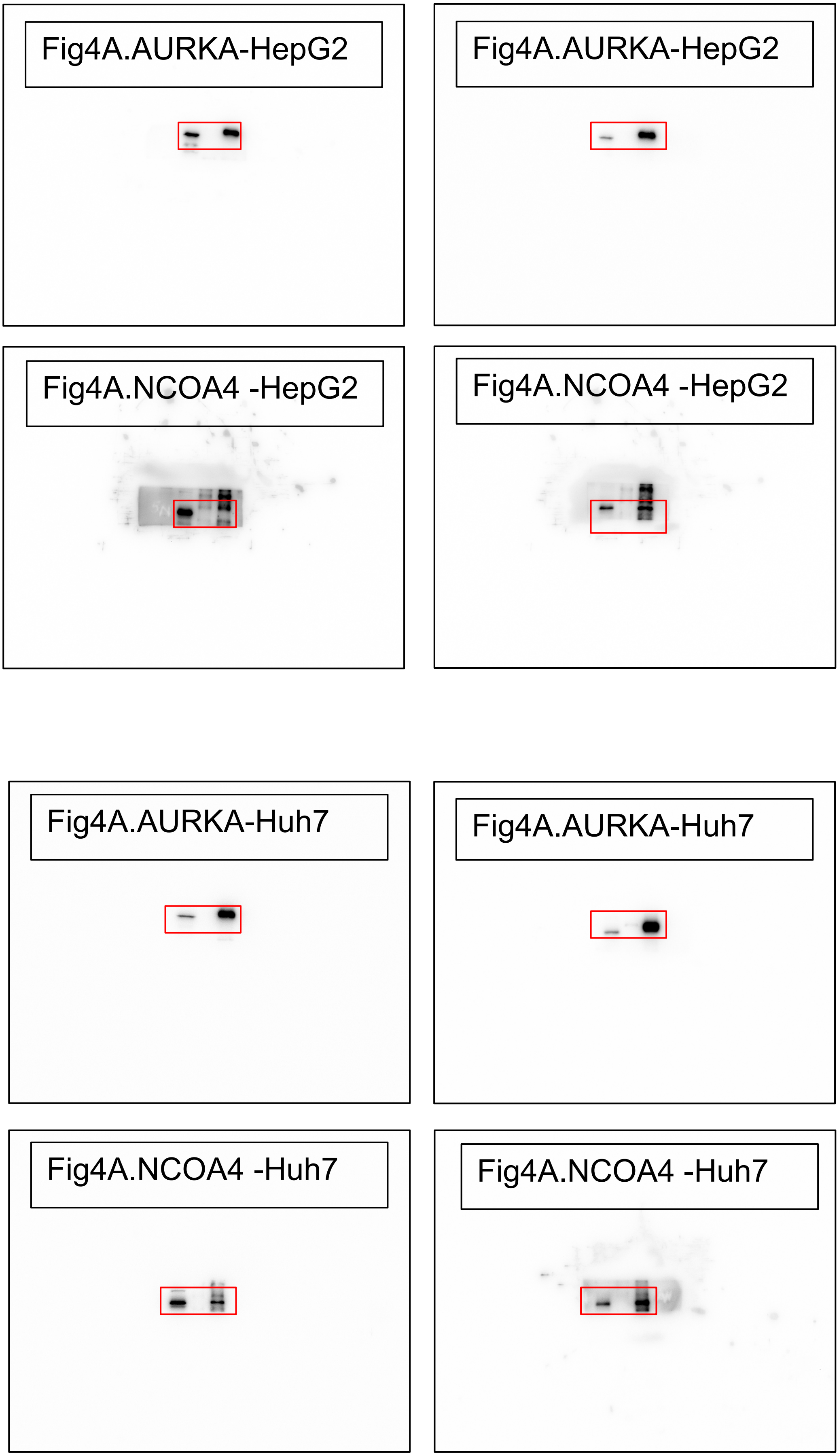

Fig4A.AURKA-HepG2
Fig4A.AURKA-HepG2
Fig4A.NCOA4 -HepG2
Fig4A.NCOA4 -HepG2
Fig4A.AURKA-Huh7
Fig4A.AURKA-Huh7
Fig4A.NCOA4 -Huh7
Fig4A.NCOA4 -Huh7

Fig.4B

Fig4B.Flag-293T

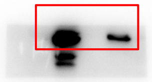

Fig4B.Flag-293T

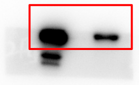

Fig4B.HA-293T

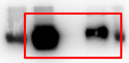

Fig4B.HA-293T

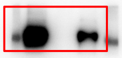

Fig.4E

Fig4E.FTH1 -HepG2

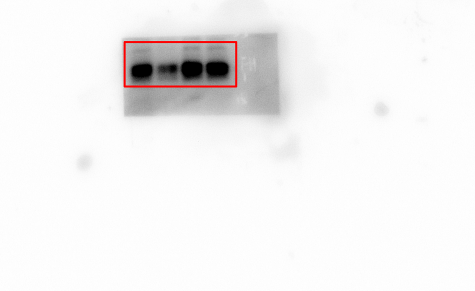

Fig4E.FTH1 -Huh7

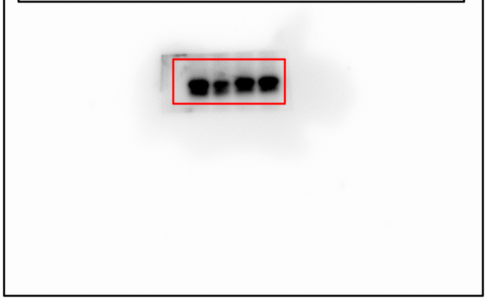

Fig4E.NCOA4 -HepG2

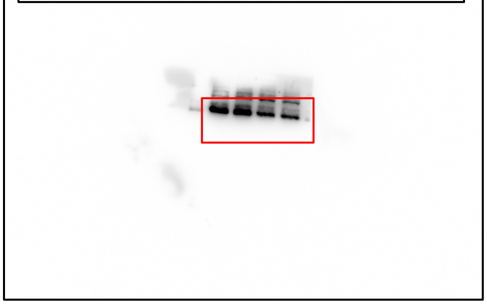

Fig4E.NCOA4 -Huh7

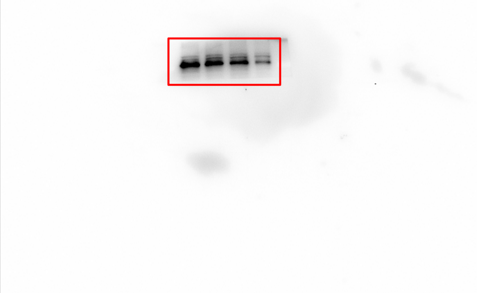

Fig4E.AURKA -HepG2

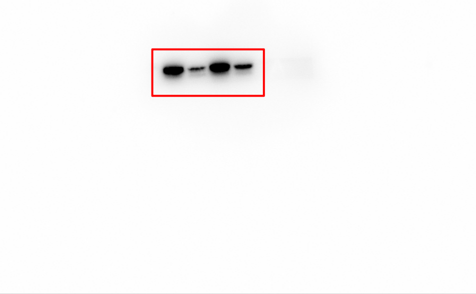

Fig4E.AURKA -Huh7

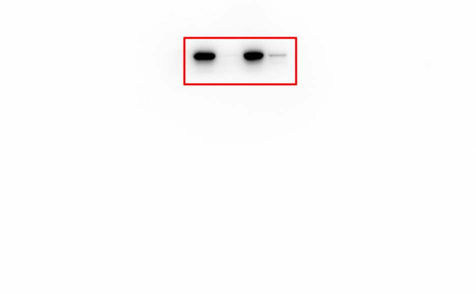

Fig4E.GAPDH -HepG2

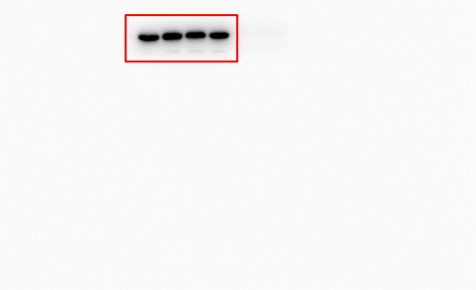

Fig4E.GAPDH -Huh7

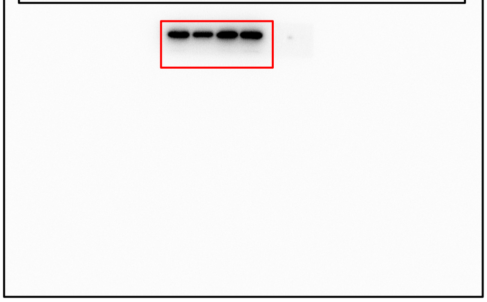

Fig.4H-HepG2

Fig4G.FTH1 -HepG2 SR

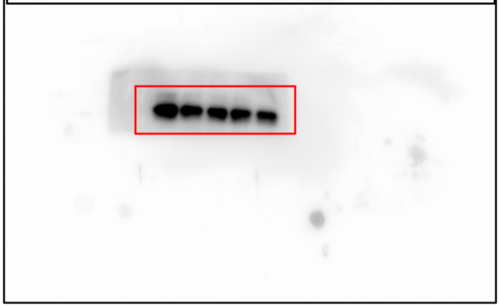

Fig4G.FTH1 -HepG2 WT

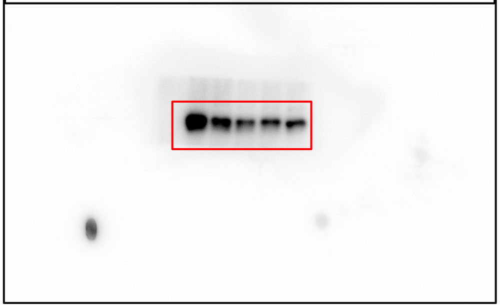

Fig4G.AURKA -HepG2 SR

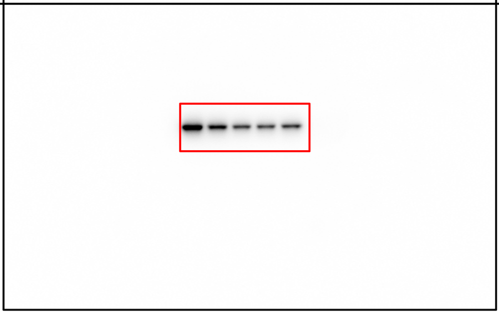

Fig4G.AURKA -HepG2 WT

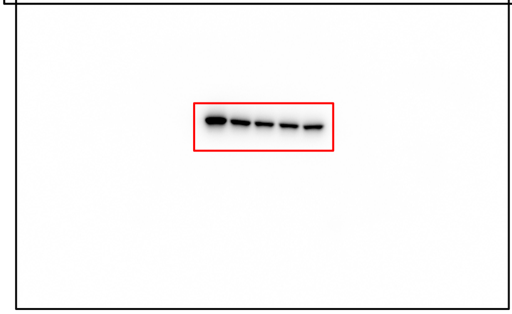

Fig4G.GAPDH -HepG2 SR

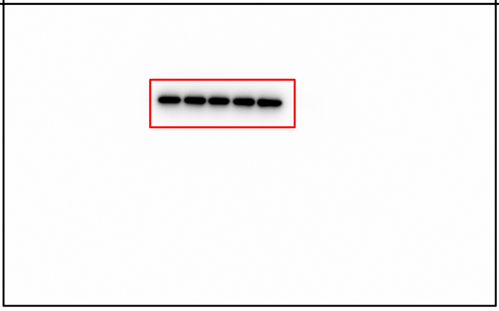

Fig4G.GAPDH -HepG2 WT

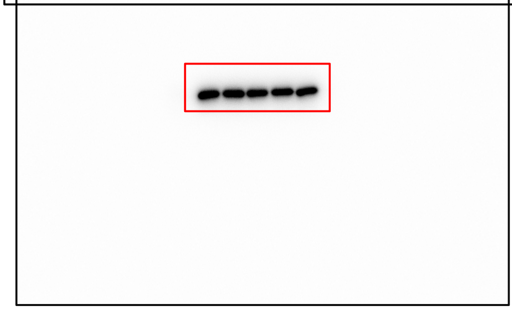

Fig.4H-Huh7

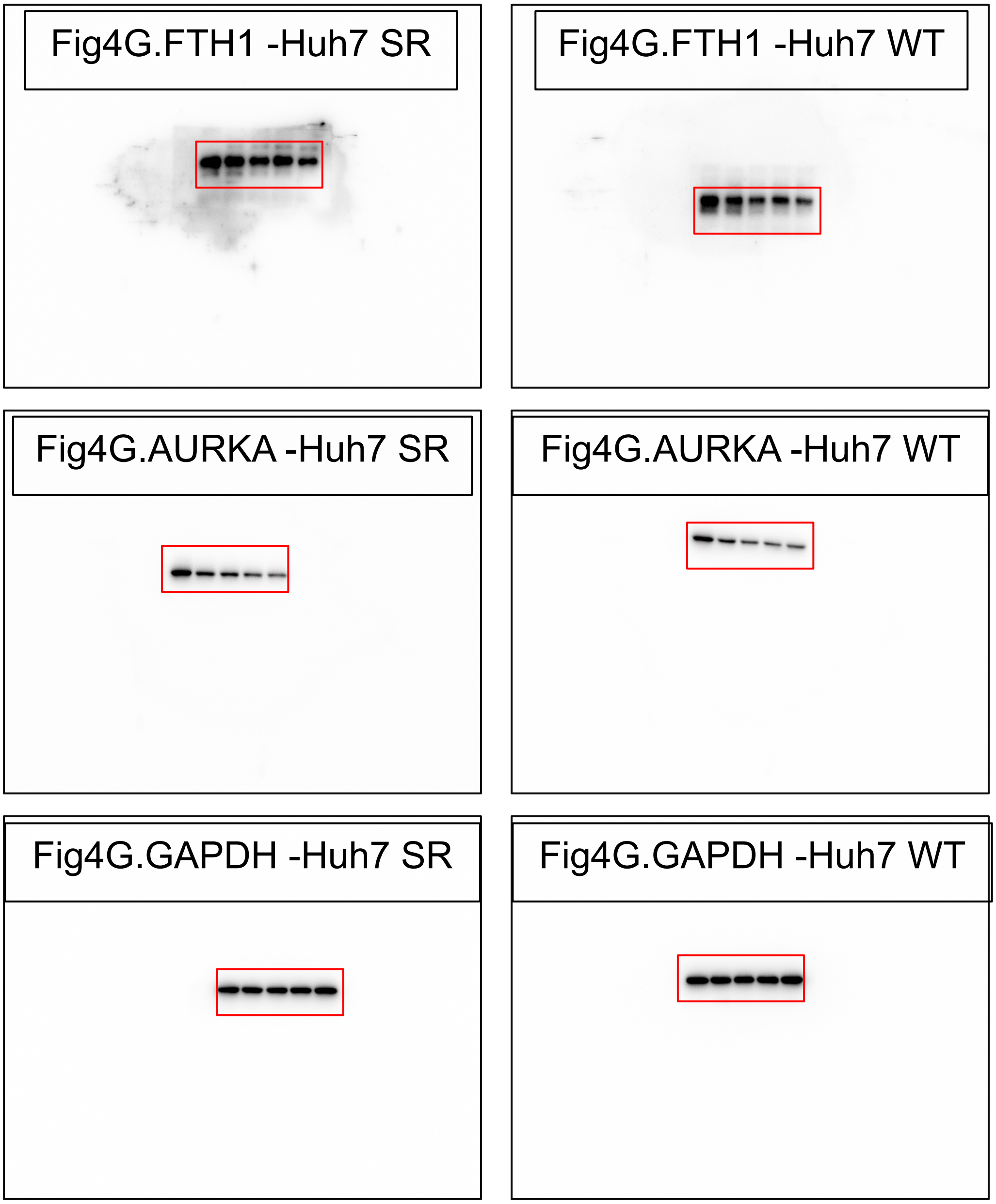
Fig4G.FTH1 -Huh7 SR
Fig4G.FTH1 -Huh7 WT
Fig4G.AURKA -Huh7 SR
Fig4G.AURKA -Huh7 WT
Fig4G.GAPDH -Huh7 SR
Fig4G.GAPDH -Huh7 WT

Fig.4J

Fig4J.FTH1 -G2 SR

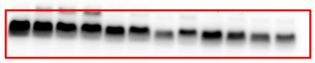

Fig4J.FTH1 -Huh7 SR

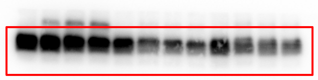

Fig4J.AURKA -G2 SR

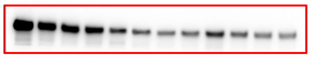

Fig4J.AURKA -Huh7 SR

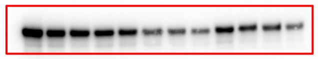

Fig4J.GAPDH -G2 SR

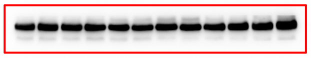

Fig4J.GAPDH -Huh7 SR

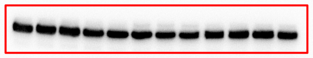

Fig.5B-HepG2

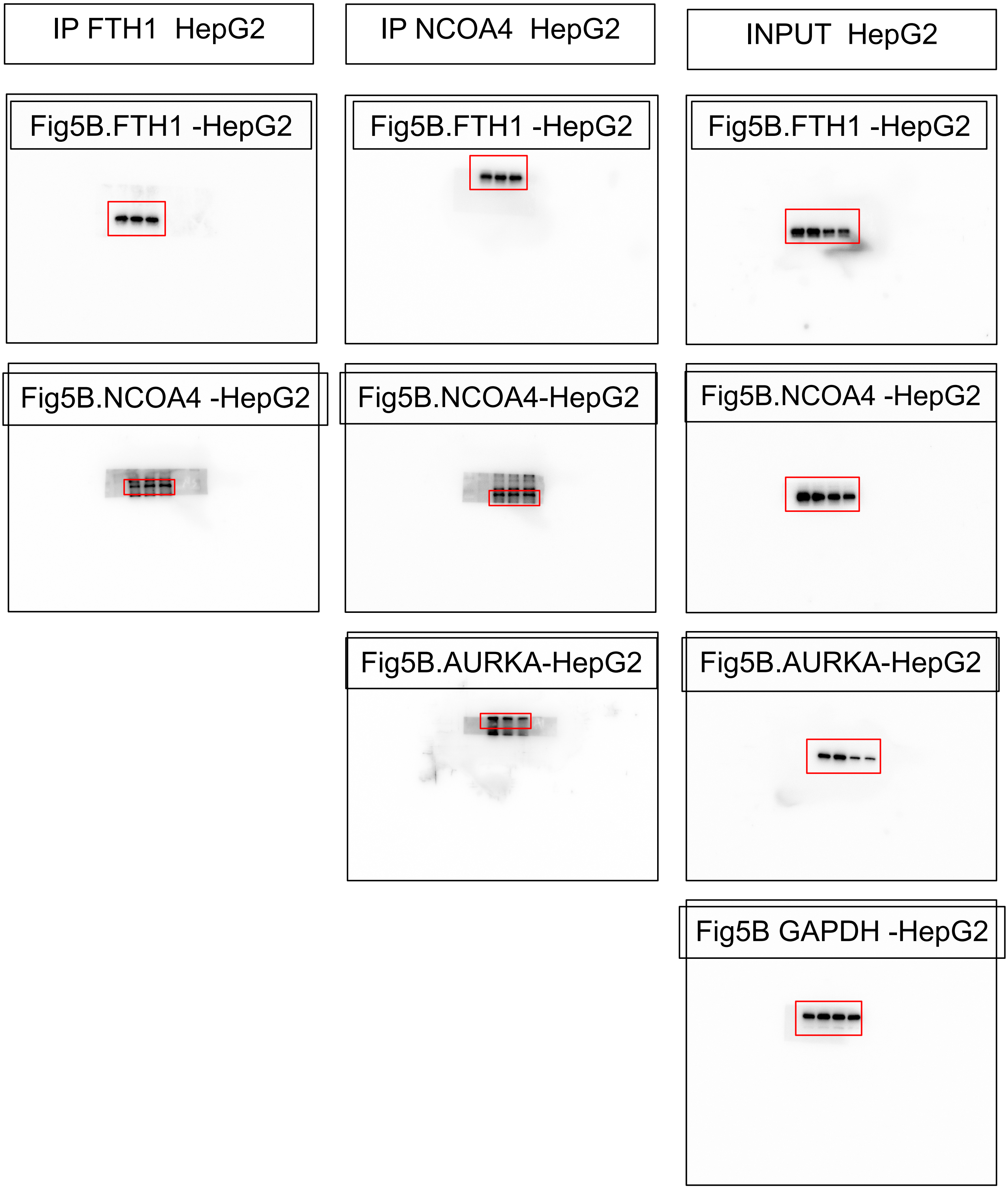

IP FTH1 HepG2
IP NCOA4 HepG2
INPUT HepG2
Fig5B.FTH1 -HepG2
Fig5B.FTH1 -HepG2
Fig5B.FTH1 -HepG2
Fig5B.NCOA4 -HepG2
Fig5B.NCOA4-HepG2
Fig5B.NCOA4 -HepG2
Fig5B.AURKA-HepG2
Fig5B.AURKA-HepG2
Fig5B GAPDH -HepG2

Fig.5B-Huh7

IP FTH1 Huh7

Fig5B.FTH1 -Huh7

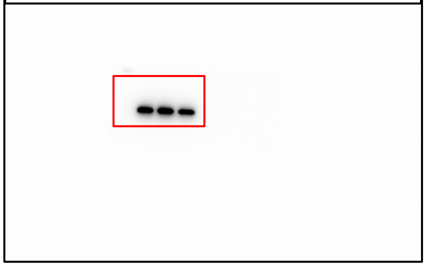

Fig5B.NCOA4 -Huh7

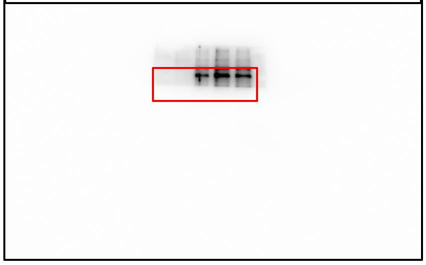

IP NCOA4 Huh7

Fig5B.FTH1 -Huh7

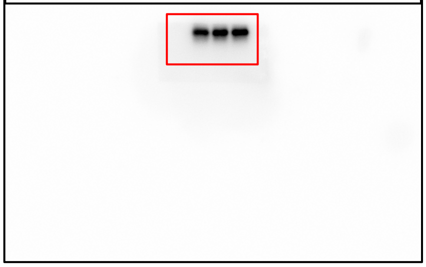

Fig5B.NCOA4 -Huh7

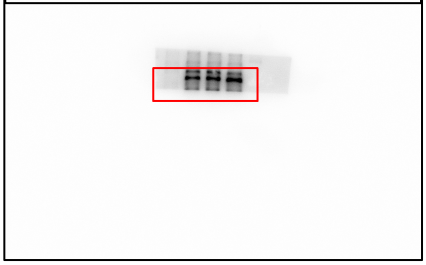

Fig5B.AURKA -HuH7

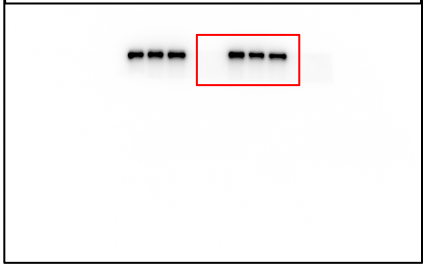

INPUT Huh7

Fig5B.FTH1 -Huh7

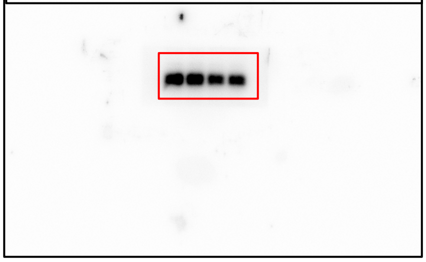

Fig5B.NCOA4 -Huh7

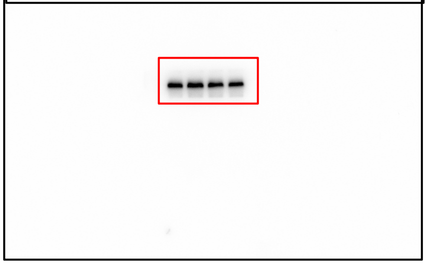

Fig5B.AURKA -HuH7

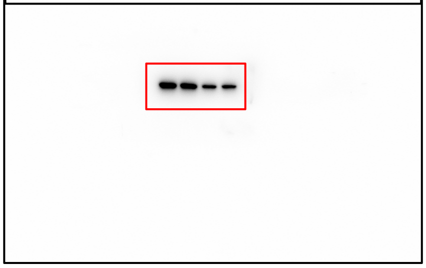

Fig5B.GAPDH -HuH7

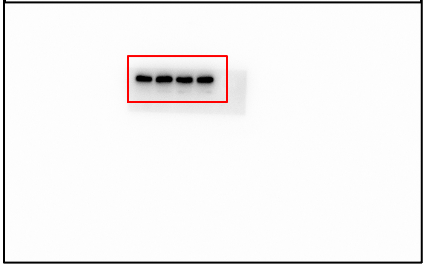

Fig.5D

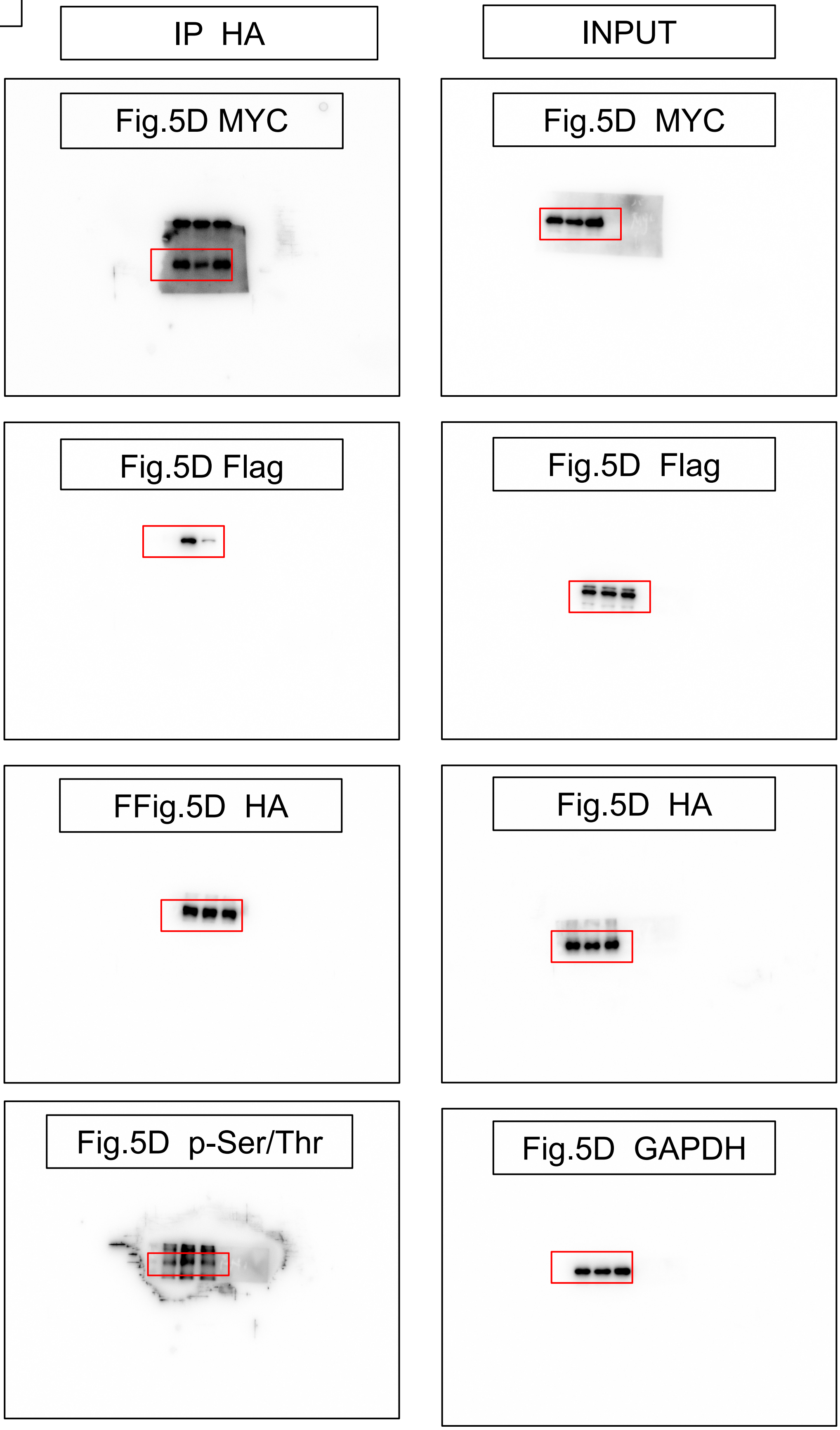
IP HA
INPUT
Fig.5D MYC
Fig.5D MYC
Fig.5D Flag
Fig.5D Flag
FFig.5D HA
Fig.5D HA
Fig.5D p-Ser/Thr
Fig.5D GAPDH

Fig.5E

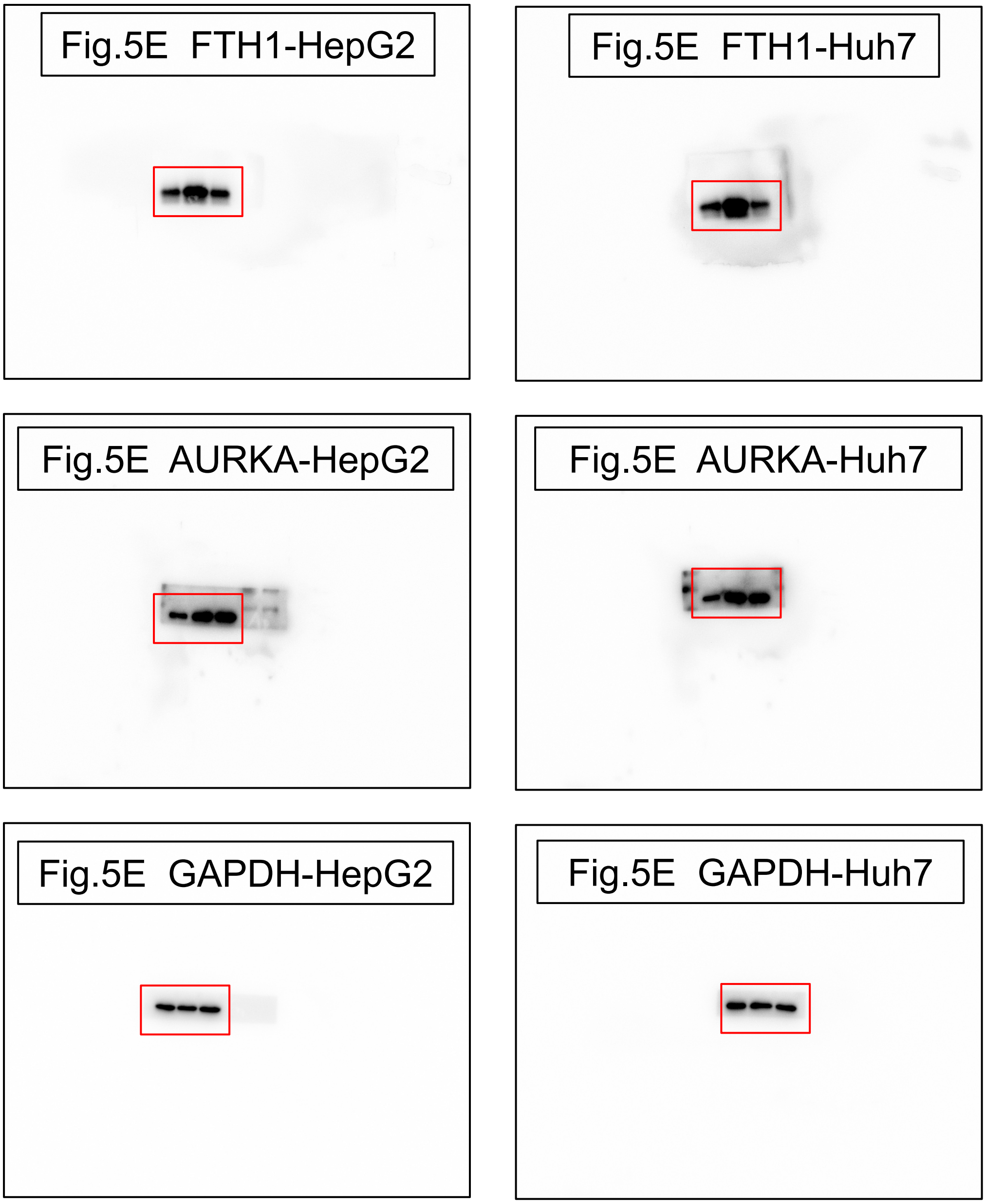
Fig.5E FTH1-HepG2
Fig.5E FTH1-Huh7
Fig.5E AURKA-HepG2
Fig.5E AURKA-Huh7
Fig.5E GAPDH-HepG2
Fig.5E GAPDH-Huh7

Fig.5H

Fig5H FTH1-HepG2

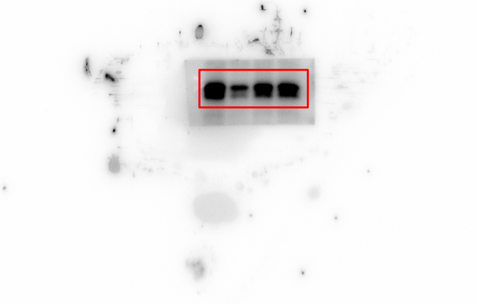

Fig5H FTH1-Huh7

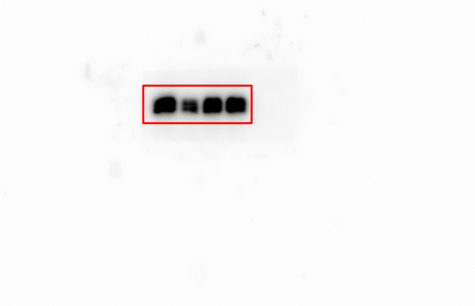

Fig5H NCOA4-HepG2

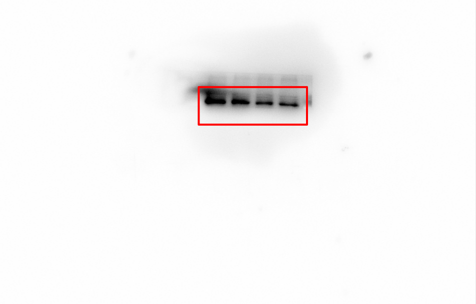

Fig5H NCOA4-Huh7

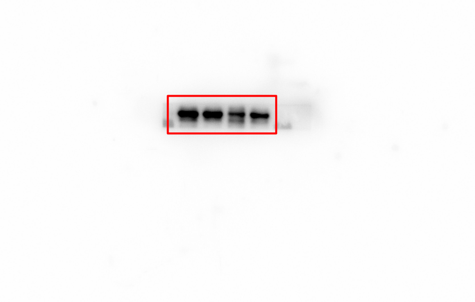

Fig5H GAPDH-HepG2

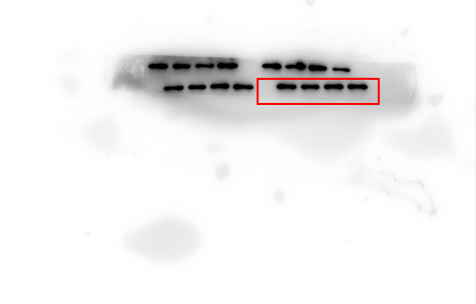

Fig5H GAPDH-Huh7

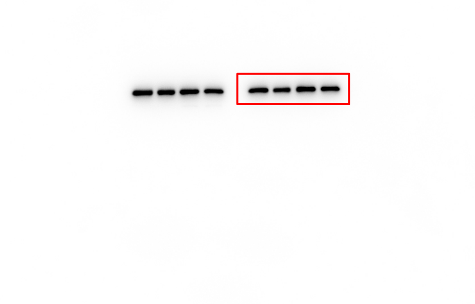

Fig.5G-HepG2

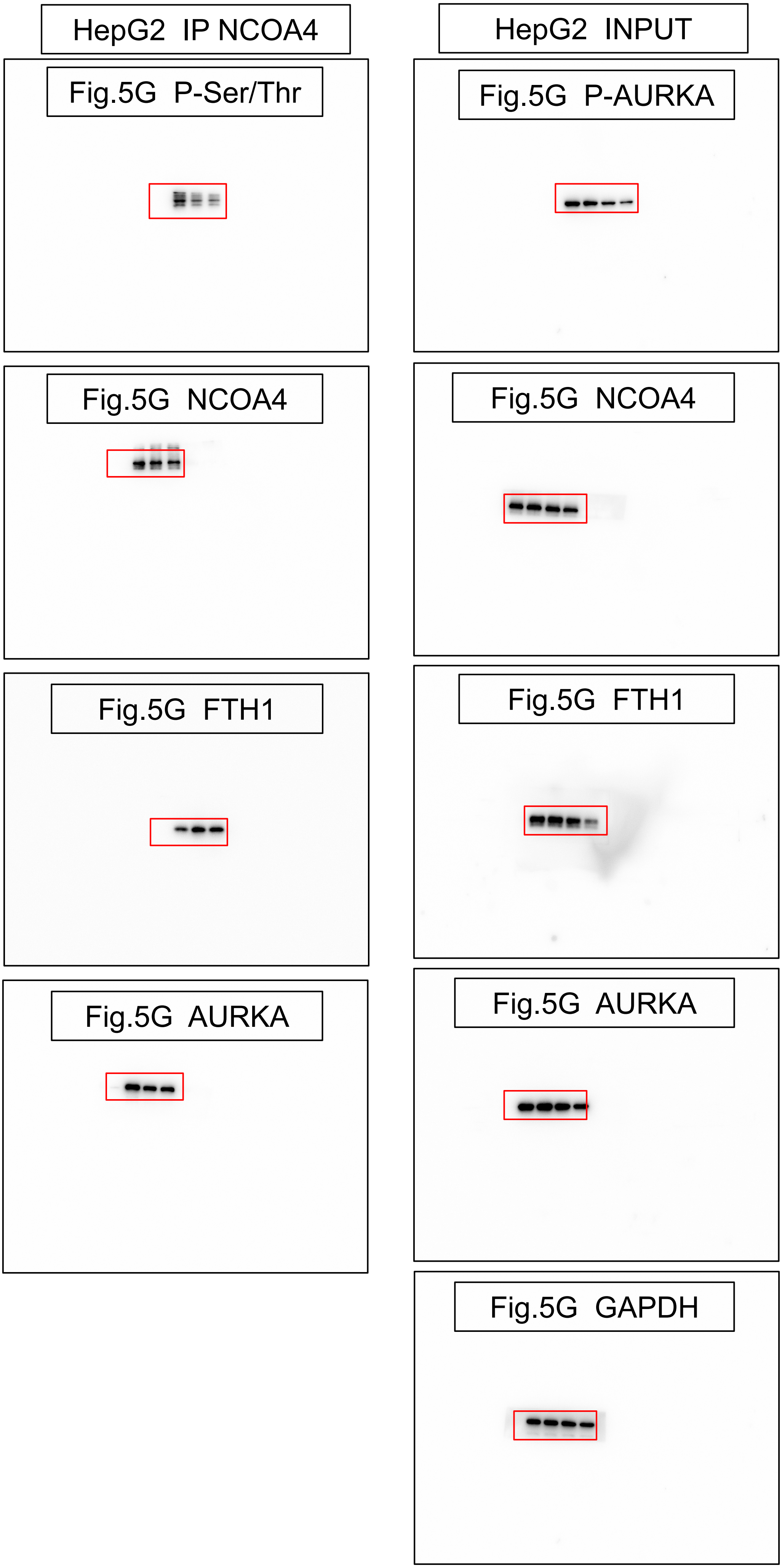
HepG2 IP NCOA4
HepG2 INPUT
Fig.5G P-Ser/Thr
Fig.5G P-AURKA
Fig.5G NCOA4
Fig.5G NCOA4
Fig.5G FTH1
Fig.5G FTH1
Fig.5G AURKA
Fig.5G AURKA
Fig.5G GAPDH

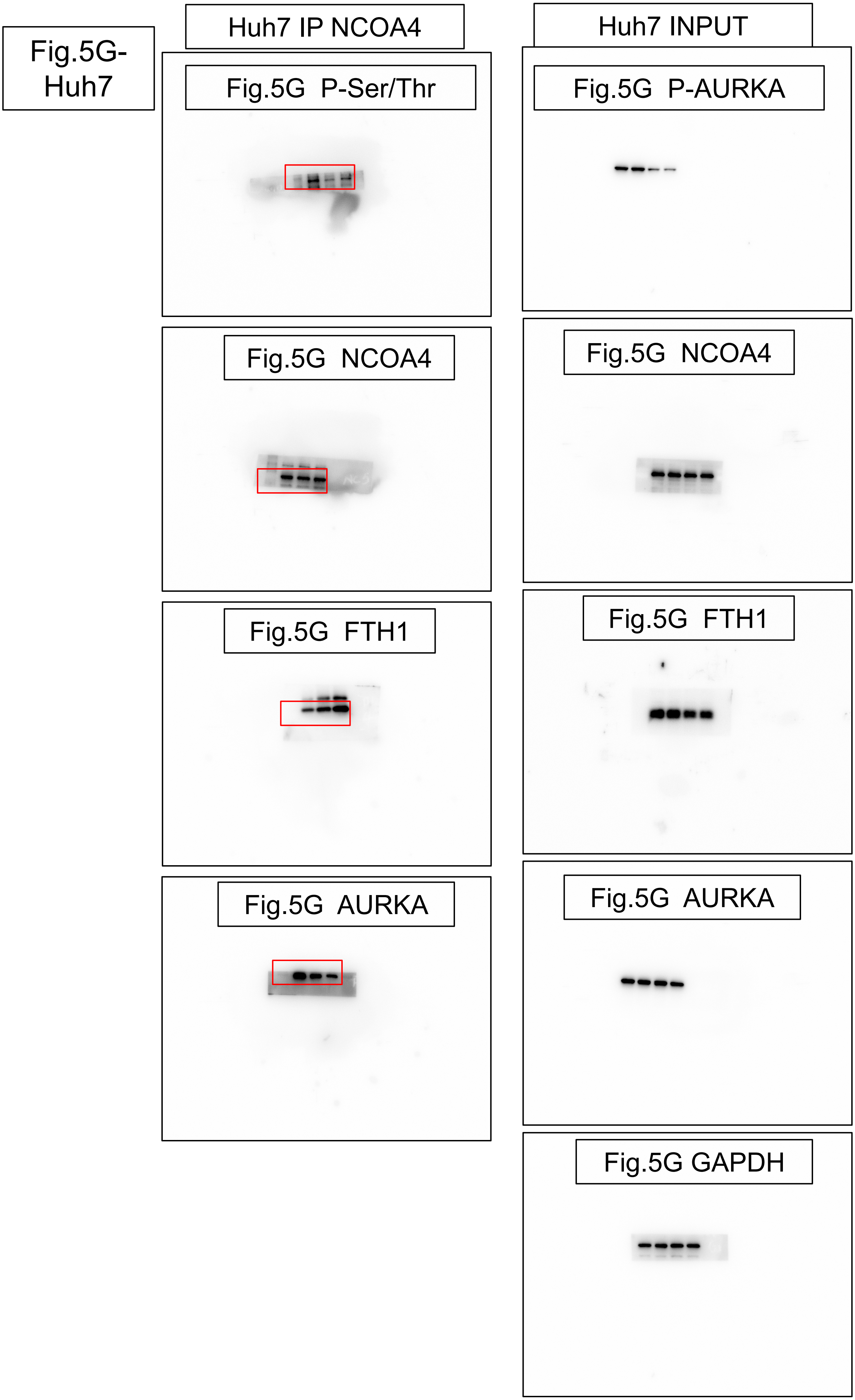

Fig.5G-Huh7
Huh7 IP NCOA4
Fig.5G P-Ser/Thr
Fig.5G NCOA4
Fig.5G FTH1
Fig.5G AURKA
Huh7 INPUT
Fig.5G P-AURKA
Fig.5G NCOA4
Fig.5G FTH1
Fig.5G AURKA
Fig.5G GAPDH

Fig.5K

IP HA

INPUT

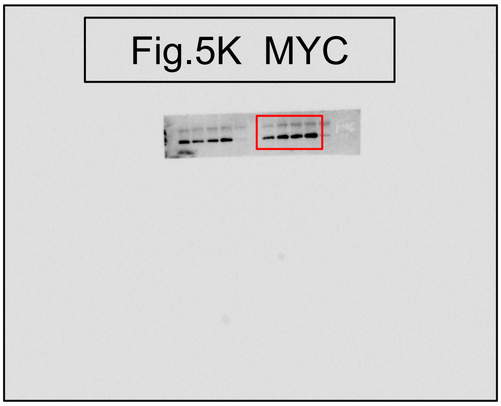

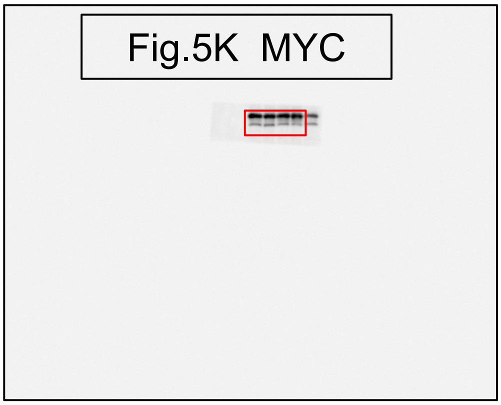

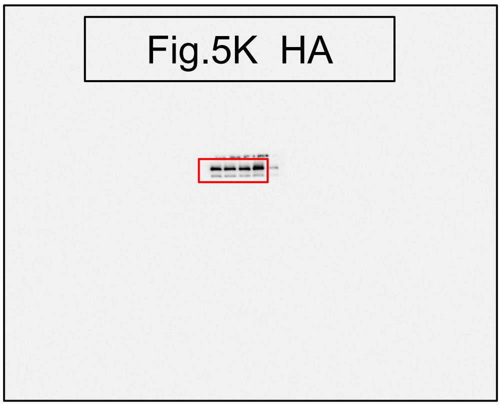

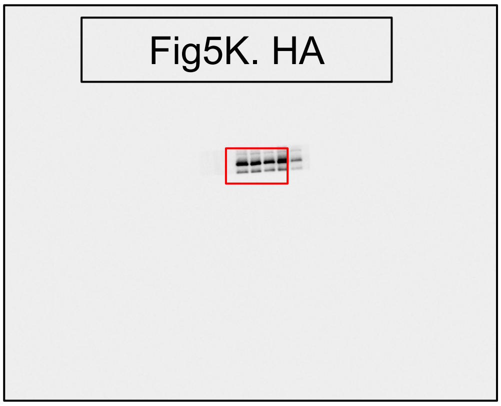

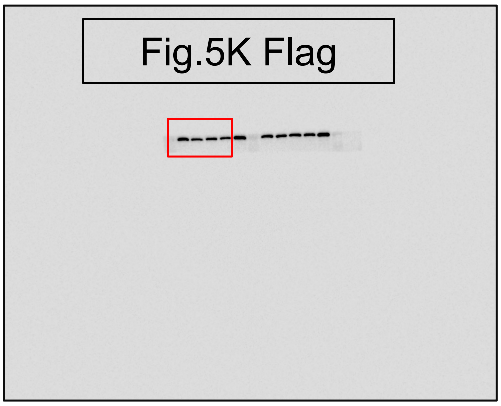

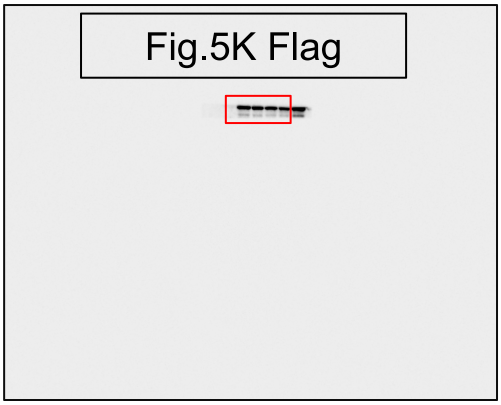

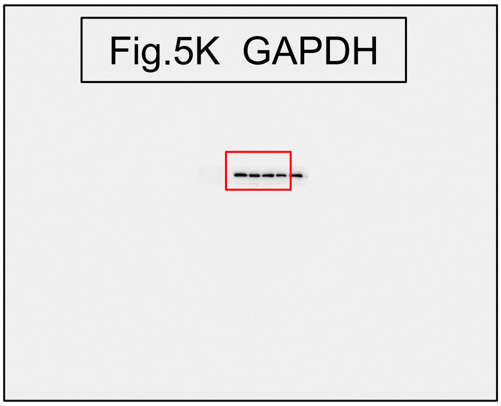

Fig.5L

Fig.5L HA

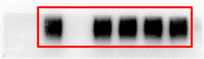

Fig.5L His

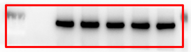

Fig.5L p- Ser

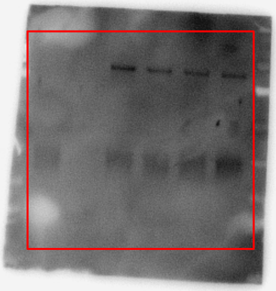

Fig.S2F

S2F.AURKA-HepG2/Huh7

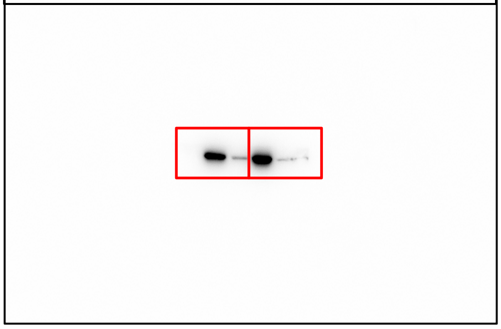

S2F.GAPDH-HepG2/Huh7

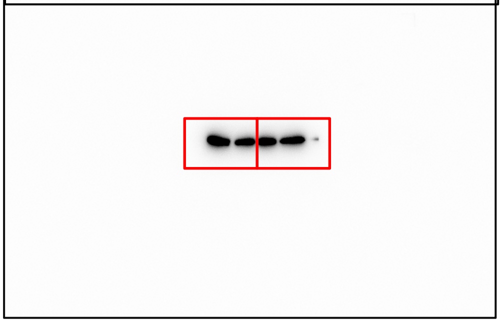

Fig.S4J

FigS.4J .DMT1 -HepG2

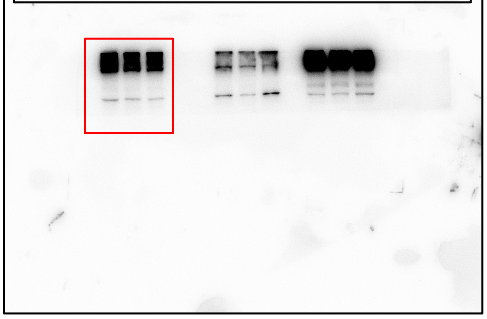

FigS.4J .DMT1 -Huh7

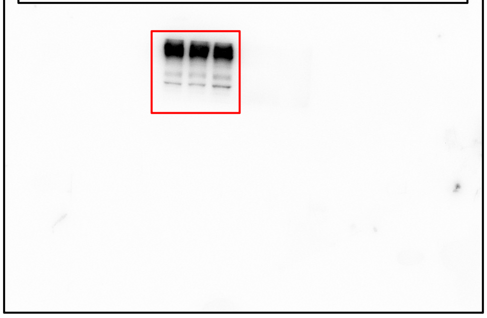

FigS.4J .TFRC1 -HepG2

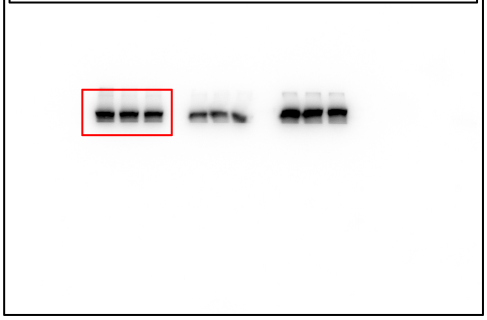

FigS.4J .TFRC1 -Huh7

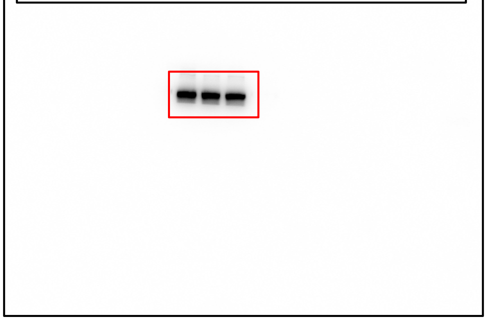

FigS.4J .AURKA -HepG2

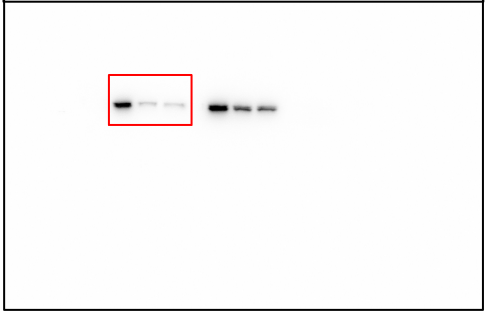

FigS.4J .AURKA -Huh7

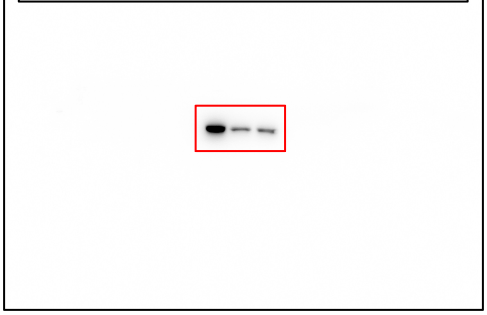

FigS.4J .GAPDH-HepG2

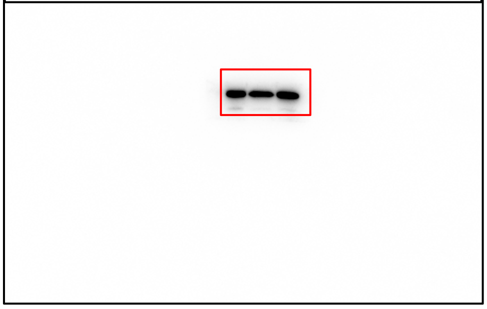

FigS.4J .GAPDH -Huh7

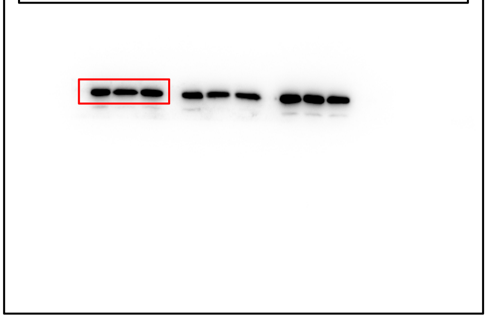

Fig.S5I

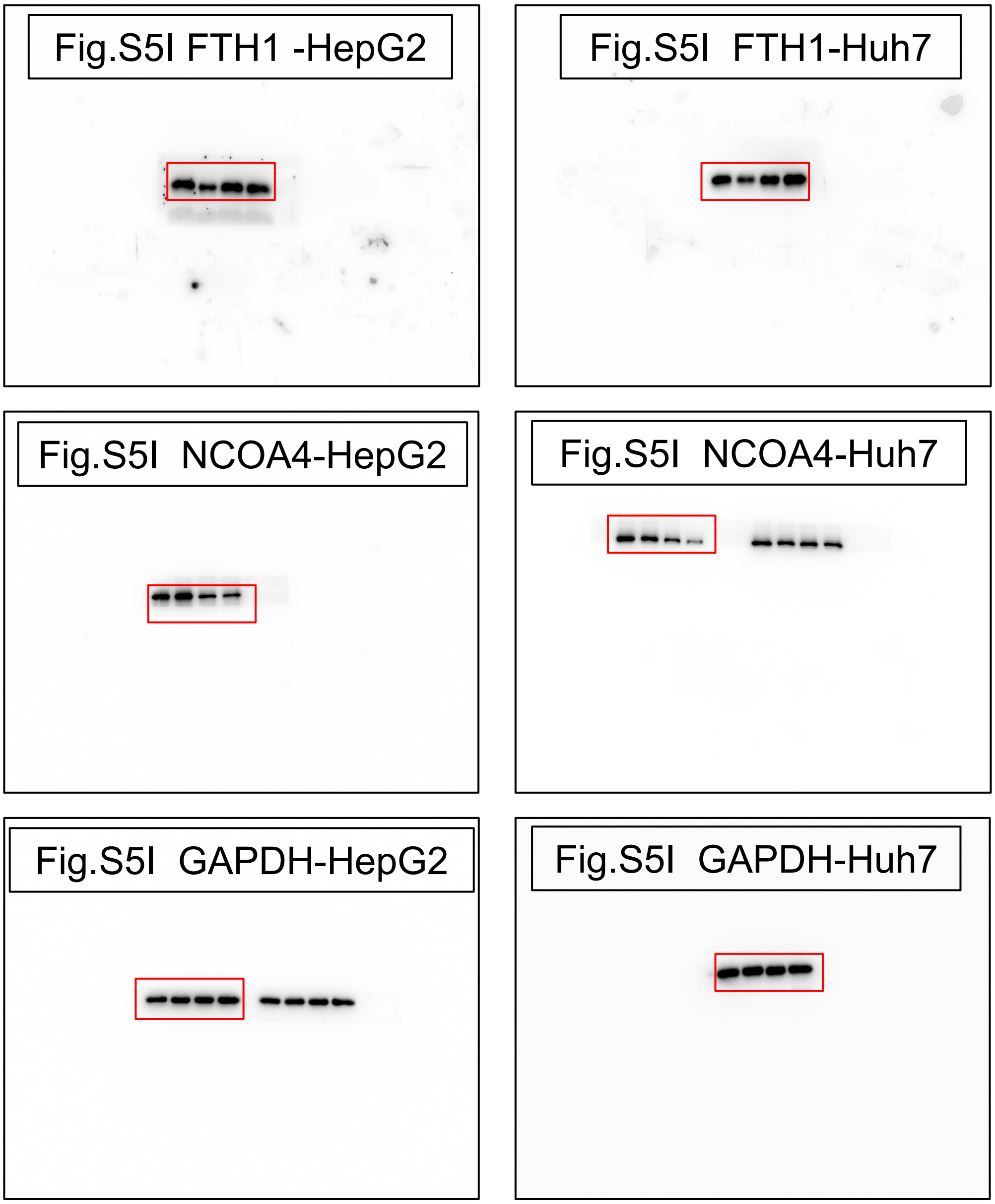
Fig.S5I FTH1 -HepG2
Fig.S5I FTH1-Huh7
Fig.S5I NCOA4-HepG2
Fig.S5I NCOA4-Huh7
Fig.S5I GAPDH-HepG2
Fig.S5I GAPDH-Huh7

Fig.S5Q

Fig.S5Q AURKA

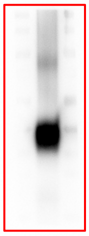

Fig.S5Q His

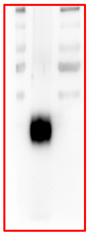

Fig.S5Q NCOA4

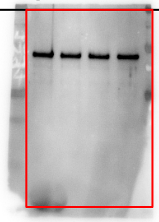

Fig.S5Q HA

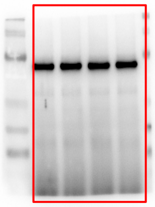

Supplement: Supplementary file 3 — Original western blots [file 41419_2026_8774_MOESM3_ESM.pdf]
